# Stabilizing lithium metal using ionic liquids for long-lived batteries

**DOI:** 10.1038/ncomms11794

**Published:** 2016-06-13

**Authors:** A. Basile, A. I. Bhatt, A. P. O'Mullane

**Affiliations:** 1 School of Applied Sciences, Applied Chemistry, RMIT University, GPO Box 2476V, Melbourne, Victoria 3001, Australia; 2 Energy Flagship, Commonwealth Scientific and Industrial Research Organisation, Clayton, Melbourne, Victoria 3169, Australia; 3 School of Chemistry, Physics and Mechanical Engineering, Queensland University of Technology, GPO Box 2434, Queensland 4001, Australia

## Abstract

Suppressing dendrite formation at lithium metal anodes during cycling is critical for the implementation of future lithium metal-based battery technology. Here we report that it can be achieved via the facile process of immersing the electrodes in ionic liquid electrolytes for a period of time before battery assembly. This creates a durable and lithium ion-permeable solid–electrolyte interphase that allows safe charge–discharge cycling of commercially applicable Li|electrolyte|LiFePO_4_ batteries for 1,000 cycles with Coulombic efficiencies >99.5%. The tailored solid–electrolyte interphase is prepared using a variety of electrolytes based on the *N*-propyl-*N*-methylpyrrolidinium bis(fluorosulfonyl)imide room temperature ionic liquid containing lithium salts. The formation is both time- and lithium salt-dependant, showing dynamic morphology changes, which when optimized prevent dendrite formation and consumption of electrolyte during cycling. This work illustrates that a simple, effective and industrially applicable lithium metal pretreatment process results in a commercially viable cycle life for a lithium metal battery.

## Abstract (web summary)

Suppressing dendrite formation at lithium anodes during cycling is critical to development of lithium battery technology. Here, the authors show that immersion of lithium electrodes in ionic liquid electrolytes prior to battery assembly produces a durable and lithium ion permeable solid-electrolyte interphase.

## Graphical abstract

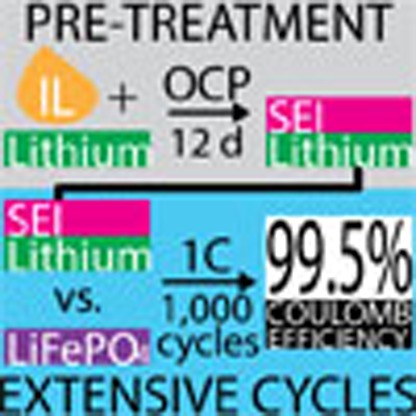

The next generations of rechargeable lithium metal anode-based battery technologies such as Li-O_2_ and Li-S have specific energies of 3,505 Wh kg^−1^ (Li-O_2_) and 2,567 Wh kg^−1^ (Li-S), respectively, which greatly exceed the current graphite anode-based technologies (typically 100–265 Wh kg^−1^). This order of magnitude increase is the breakthrough needed for applications such as electric vehicles where concerns of ‘range anxiety' are negated; in principle, these vehicles could compete with traditional petrol vehicles in terms of drive range per charge. Unlike graphite, lithium metal anodes do not intercalate lithium ions, instead requiring repetitive stripping and plating of the lithium metal surface. This causes volumetric and morphological changes that can lead to cycling instability. The inelastic solid–electrolyte interphase (SEI) formed during cycling cannot compensate for these morphological changes, and as a result is in a continual state of repair. This generally leads to low Coulombic efficiency (CE) and short cycle life because of electrolyte consumption and loss of lithium through cyclic SEI production.

This highlights a significant issue concerning the safe cyclability of lithium metal, that is, difficulty in controlling the thermodynamic instability and reactivity of lithium towards organic electrolytes^1^,^2^. These factors contribute to the classic literature examples of lithium metal battery failure, which requires avoiding large Li dendrite^3^,^4^ and non-uniform SEI formation that typically results in thermal runaway via the creation of a short circuit^4^,^5^,^6^,^7^. Therefore, it is critical that there is not only further development in understanding SEI formation^5^,^8^,^9^ but also a significant research effort into tailoring the SEI that is transferable into devices that can be practically realized.

Room-temperature ionic liquid (RTIL) electrolytes are potentially a successful alternative to conventional organic solvent-based electrolytes^10^ and have shown promising results^11^,^12^,^13^. In particular, RTILs composed of the bis(fluorosulfonyl)imide [FSI]^−^ anion are attracting significant attention^14^,^15^. The RTIL comprising the pyrrolodinium cation and *N*-propyl-*N*-methylpyrrolidinium bis(fluorosulfonyl)imide anion, namely ([C_3_mPyr^+^][FSI^−^]), possesses low viscosity^16^ and has been associated with delayed Al corrosion at the cathode^17^,^18^. Previously, Budi *et al.* and other reports noted that the SEI formed through chemical pathways using [C_3_mPyr^+^][FSI^−^] provides a smooth morphology that suppresses dendrite formation in symmetrical Li|electrolyte|Li cells, which is a critical prerequisite for secondary cell cycling^19^,^20^.

Ideally, the prospect of tuning the electrolyte to affect a beneficial change in electrode performance^21^, rather than modifying the lithium electrode via complicated and tedious surface engineering with nanostructures^22^,^23^,^24^, would in principle allow a pathway towards simplified and cheaper battery construction. This approach could readily be translated to existing manufacturing processes and therefore accelerate the development of next-generation energy-storage solutions. We herein report on facile SEI formation via a simple chemical interaction between Li^0^ and [C_3_mPyr^+^][FSI^−^] mixtures utilizing LiFSI, LiPF_6_ and LiAsF_6_ salts. Remarkably, we observe extensive Li|electrolyte|Li cell cycling at 0.1 and 1.0 mA cm^−2^ for symmetrical cells pretreated using LiFSI and LiAsF_6_ salts, respectively, dissolved in [C_3_mPyr^+^][FSI^−^], with no evidence of dendrite formation even after 2,500+ hours cycling time. Full Li|electrolyte|LiFePO_4_ batteries were also evaluated, and these batteries display extensive safe cycling at a rate of 1 C, achieving 1,000 cycles with a CE greater than 99.5% after pretreatment, comparable to or, in certain cases, outperforming current commercial 18,650 Li ion batteries^25^. In addition, this approach is beneficial for cells that are left on the shelf, where a cell that was quiescent for 330 days is shown to cycle effectively with >95% CE.

## Results

### Time-dependent SEI formation

Our previous work showed that Li reacts with [C_3_mPyr^+^][FSI^−^] to form an SEI layer, and that the application of a potential is not required to observe significant changes on the electrode surface^19^. However, on addition of Li salts to the RTIL there is a dramatic difference in both the reaction rate and composition of the SEI, which has significant implications for the performance of symmetrical and full cells. This initial interaction between the electrolyte and Li anode, occurring on construction of any cell, is a critical factor that can in fact be tailored to increase cyclability, safety and CE, and is the focus of this study that uses [C_3_mPyr^+^][FSI^−^] as the ionic liquid.

**Figure 1:**
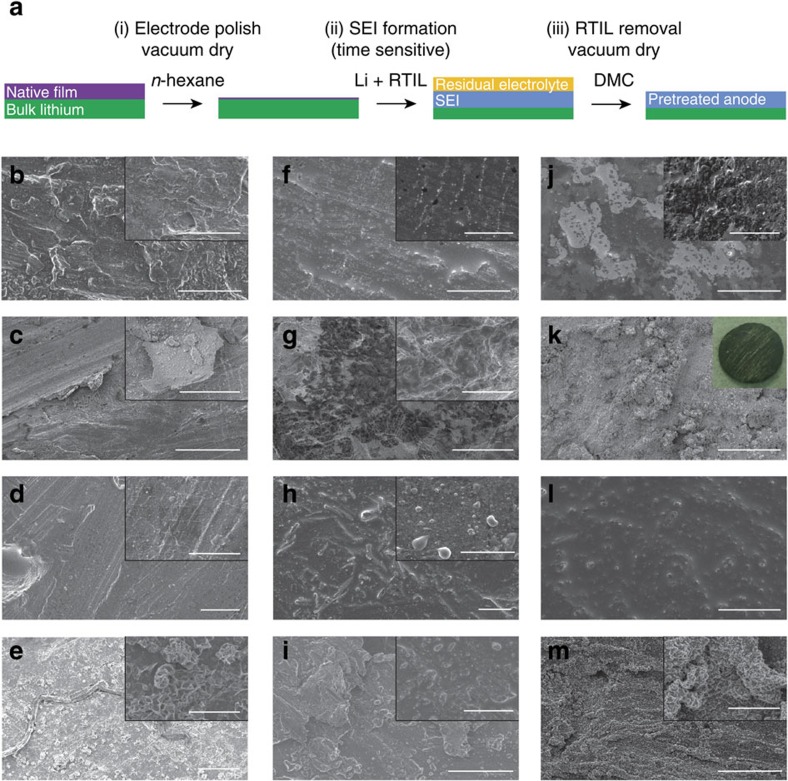
Scanning electron micrographs of the lithium morphology after pretreatment. (**a**) Facile process for the preparation of SEI materials via chemical pretreatment with ionic liquid electrolyte. Micrographs revealing the dynamic morphology changes at lithium using LiFSI/[C_3_mPyr^+^][FSI^−^] electrolyte at time periods of (**b**) 4 h, (**c**) 7 d, (**d**) 12 d and (**e**) 18 d. As above, for the LiPF_6_/[C_3_mPyr^+^][FSI^−^] electrolyte after (**f**) 4 h, (**g**) 7 d, (**h**) 12 d and (**i**) 18 d, and while using LiAsF_6_/[C_3_mPyr^+^][FSI^−^] electrolyte after (**j**) 4 h, (**k**) 7 d, (**l**) 12 d and (**m**) 18 d. Scale bars, 50 μm (inset scale bars, 10 μm). Additional time periods available in Supplementary Fig. 2.

The facile lithium pretreatment methodology is shown in Fig. 1a, and the observed morphological changes that occur for the LiFSI/[C_3_mPyr^+^][FSI^−^] electrolyte system after the reaction time of 4 h, 7, 12 and 18 days are illustrated in Fig. 1b–e. Moreover, shown are micrographs of a lithium surface pretreated with LiPF_6_/[C_3_mPyr^+^][FSI^−^] (Fig. 1f–i) and LiAsF_6_/[C_3_mPyr^+^][FSI^−^] (Fig. 1j–m) for the same time periods. These micrographs show the diverse topology that can be realized by tuning reaction time and/or electrolyte composition. In all cases there are dramatic changes in the Li surface compared with the relatively smooth pristine metal (Supplementary Fig. 1).

The SEI surface-morphology changes on salt addition are significantly different to that observed for neat [C_3_mPyr^+^][FSI^−^]^19^. Interestingly, inclusion of a Li salt results in accelerated changes, which are attributed to the anion. After 4 h of pretreatment with LiFSI/[C_3_mPyr^+^][FSI^−^] (Fig. 1b) the lithium surface contains residual electrolyte with numerous SEI sites. For foils pretreated for 7 days, the surface becomes rough and exfoliated (Fig. 1c and inset). After 12 days, the smoothened surface exhibits ordered SEI deposition sites preferentially arranged into lines of equal spacing of *ca* 0.25–11.0 μm (Fig. 1d). These ordered deposition sites eventually cluster to form ribbons (up to 500 μm in length) after 18 days (Fig. 1e). Honeycomb structures were also observed, dissimilar to those reported for LiBF_4_/[C_3_mPyr^+^][FSI^−^]^14^.

The LiPF_6_/[C_3_mPyr^+^][FSI^−^] electrolyte reacts producing a rough surface with evidence of small deposits on the surface after 4-h interaction (Fig. 1f). Figure 1g shows that additional species (dark areas) dominate the surface and is attributed to an exfoliation process. After 12 days, a thick film of deposition products is formed (Fig. 1h), with rod-like features and surface electrolyte coverage. By 18 days, these resolve into discreet-layered islands of deposition products (Fig. 1i).

For the LiAsF_6_/[C_3_mPyr^+^][FSI^−^] electrolyte there are three distinct regions formed after 4 h (Fig. 1j), showing deposition sites situated within both the darkest and lightest regions. Medium contrast regions contain adhered residual electrolyte and rough areas are also observed (inset in Fig. 1j). By 7 days of interaction time, there is complete coverage of Li by coral structures (Fig. 1k); unlike the LiPF_6_/[C_3_mPyr^+^][FSI^−^] pretreatment this structure dominates and alters the entire morphology. Optically, the lithium foil shows a tarnish over the metal (inset in Fig. 1k—typical LiAsF_6_/[C_3_mPyr^+^][FSI^−^]-pretreated foil), whereas all other electrolyte-treated samples retain a reflective surface. After 12 days (Fig. 1l), the surface is dominated by electrolyte adhesion and is presumed to envelop the structures observed after 7 days. After 18 days, the electrolyte and breakdown products become part of the structure. A consolidation of both the deposition products and the electrolyte forms a coral superstructure with complete surface coverage (Fig. 1m). It is clear that the 12-day time point shows a common trend for all electrolytes in that a smoother surface is formed that does not contain as many protrusions and coral-like structures that are present at other times. Scanning electron microscopy (SEM) images of additional reaction time periods of 4, 10, 14 and 16 days (Supplementary Fig. 2) further illustrate the gradual change of the Li surface. This time-dependent morphological change could have significant implications for battery performance, as this layer will be the initial seed layer that dictates the Li ion ingress/egress during initial cell cycling.

### Surface chemistry of SEI via FTIR and XPS

The composition of the diverse SEI structures formed on the lithium surface after pretreatment was identified utilizing a combination of Fourier transform infra-red (FTIR) and X-ray photoelectron spectroscopy (XPS). A full description of the FTIR peak assignments and XPS data is provided in Supplementary Note 1. Table 1 presents the XPS spectral data assignments recorded for lithium foils after pretreatment with each of the electrolytes after 7 days.

In the case of pretreatment with [C_3_mPyr^+^][FSI^−^], the SEI consists of LiF, LiOH and SO_2_F species from breakdown of the RTIL, accompanied by generation of SO_2_ gas and surface-bound [C_3_mPyr^+^][FSI^−^]^19^. On addition of LiFSI, there is a rise in the intensity of infrared (IR) bands at *ca*. 1,106 and 1,170 cm^−1^, suggesting a greater amount of anion breakdown products (Supplementary Table 1). The SEI now changes and consists of LiF, LiSO_2_F, NSO^−^ species, LiOH and Li_2_S via reduction of the anion (Table 1). Notably, this breakdown process occurs without evolution of SO_2_ gas, which illustrates the beneficial properties of a mineral SEI^13^. In addition, the increased concentration of [FSI]^−^ promotes the breakdown of the cation to propyl pyrrolidine, with reduction taking place via a Hofmann β-elimination (Supplementary Fig. 3), and was observed via FTIR^26^,^27^,^28^. In addition, FTIR measurements reveal that the SEI also contains surface-bound cation and anion species as well as Li_2_CO_3_ from the native lithium foils.

The conclusion for this mechanism of SEI formation is detailed in the data in Table 1, which illustrates a Li 1*s* peak encompassing the responses for both LiF (56.1 eV) and Li-O (55.3 eV), while the F 1*s* spectrum validates the assignment with a response measured at 685.1 eV. The F 1*s* spectrum also suggests the presence of LiSO_2_F with a band at 688.3 eV, associated with the doublet at 170.7 and 169.9 eV (S 2*p*), 55.3 eV (Li 1*s*) and 533.2 eV (O 1*s*). This response is highly likely after further reduction of the stable ^·^SO_2_NSO_2_F^−^ radical as validated ref. 13. In that report the oxidation, reduction and subsequent fragmentation of the [FSI]^−^ at a lithium surface is measured via radiolysis and matrix-isolation electron paramagnetic resonance. That work clarifies the ability of the highly stable ^·^SO_2_NSO_2_F^−^ radical ion, which persists after LiF formation, to allow further reduction^13^:


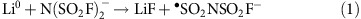



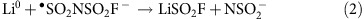


This enables SEI formation to take place without the evolution of SO_2_ gas, which compromises the beneficial properties of a mineral SEI and is consistent with the report of Philippe *et al.* on using LiFSI at nanosilicon electrodes^29^; however, cleavage of the S–N bond was not described.

The C 1*s* spectrum contains a small peak at 286.3 eV, confirming the presence of the cation at the lithium surface (C–N^+^) and was reciprocated in the N 1*s* peak at 403.0 eV. The presence of Li_2_CO_3_ is also confirmed with peaks at 55.3 eV (Li 1*s*), 289.8 eV (C 1*s*) and 533.2 eV (O 1*s*), which is known to reside in the native lithium foils^30^. The peak observed at 160.6 eV is indicative of Li_2_S, and has been detected in other studies of RTIL interaction at lithium metal^31^,^32^,^33^.

The reduction of products from equation (2) may take place on lithium as follows^13^


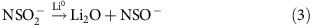



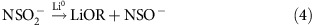


Li_2_O is not detected via XPS measurements, ruling out equation (3); however, the LiO^−^ precursor is observed as LiOH-sharing responses with Li_2_CO_3_ discussed above, giving confidence to equation (4). The thiazate anion may then be assigned to the N 1*s* peak at 400.1 eV (Table 1) as the final [FSI]^−^ moiety, which is at a similarly low abundance to the cation N 1*s* peak at 403.0 eV.

Similar breakdown products comprise the LiAsF_6_/[C_3_mPyr^+^][FSI^−^]-pretreated SEI. Namely, propyl pyrrolidine via Hofmann β-elimination of the cation, LiF, LiSO_2_F, NSO^−^ and Li_2_S from anion breakdown, Li_2_CO_3_ from the native lithium metal and surface-bound anion and cation. However, the absence of bands in the As 3*d* spectra is indicative of As not taking part in SEI formation. The tarnished surface observed is usually an indication of the well-known arsenic–oxygen polymer ‘brown film'^34^. Interestingly, FTIR data (Supplementary Table 2) only show evidence for this at earlier time periods, suggesting that this reaction is not proceeding to completion because of an absence of polymerizing precursors^35^.

Finally, for treatment with LiPF_6_/[C_3_mPyr^+^][FSI^−^] (Supplementary Table 3), the SEI comprises LiSO_2_F, NSO^−^, surface-bound cations and anions and Li_2_S. For this pretreatment, a lower quantity of LiF, LiOH and Li_2_CO_3_ was observed via energy-dispersive spectroscopy. The most notable difference for this system is the formation of methyl pyrrolidine. The Hofmann β-elimination discussed above may occur via three different pathways. The dominant pathway leads to ring cleavage towards the propyl pyrrolidine species. However, with LiPF_6_ addition, the less preferred pathway now leads to the formation of methyl pyrrolidine. Finally, breakdown of the 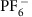 anion to form PF_*x*_ species is also found. Therefore, the XPS data indicate that the electrolyte containing LiPF_6_ has a markedly different composition to the other electrolytes, which may have an impact on the performance of any cell that incorporates this electrolyte. Overall, the SEI products formed for each electrolyte system are:

- LiFSI/[C_3_mPyr^+^][FSI^−^]: LiF, LiOH, LiSO_2_F^−^, NSO^−^, [C_3_mPyr]^+^, [FSI]^−^, propyl pyrrolidine, Li_2_CO_3_ and Li_2_S
- LiPF_6_/[C_3_mPyr^+^][FSI^−^]: LiF, PF_*x*_, LiOH, Li_2_CO_3_, methyl pyrrolidine, NSO^−^, [C_3_mPyr]^+^ and [FSI]^−^
- LiAsF_6_/[C_3_mPyr^+^][FSI^−^]: Li_2_CO_3_, propyl pyrrolidine, LiF, Li_2_SO_2_F, NSO^−^, [C_3_mPyr]^+^ and [FSI]^−^

Therefore, the different surface morphologies seen as a result of exposure to different Li salts (Fig. 1) can be attributed to the different species that constitute the SEI. However, it is quite remarkable that the kinetics of the process are similar, given the appearance of a smooth compact SEI for each system at the 10–12-day mark.

### Morphological changes after extended cycling of untreated foils

**Figure 2:**
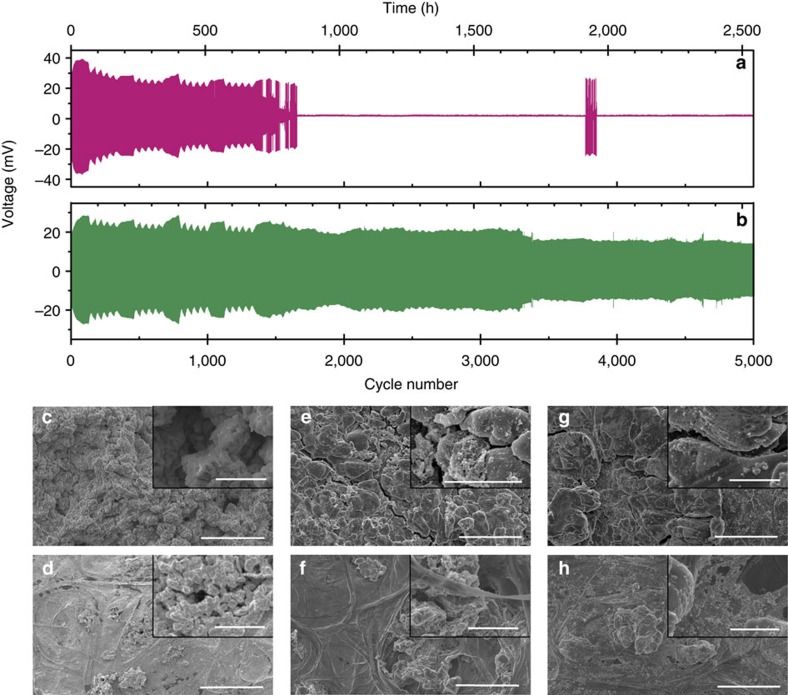
Characterization of Li-symmetrical cells during 5,000 cycles at 0.1 mA cm^−2^. Representative voltage–time plot for long-term cycling of a symmetrical cell containing (**a**) LiPF_6_/[C_3_mPyr^+^][FSI^−^] or (**b**) LiAsF_6_/[C_3_mPyr^+^][FSI^−^] electrolytes. SEM micrographs displaying dendrite suppression for the (**c**) anode and (**d**) separator of the LiFSI/[C_3_mPyr^+^][FSI^−^] cell; (**e**) anode and (**f**) separator for the LiAsF_6_/[C_3_mPyr^+^][FSI^−^] cell; (**g**) anode and (**h**) separator for the LiPF_6_/[C_3_mPyr^+^][FSI^−^] cell. Scale bars, 50 μm (inset scale bars, 10 μm).

Figure 2 shows the voltage–time (V–t) profiles for Li|electrolyte|Li cells using untreated lithium cycled at 0.1 mA cm^−2^. The cell containing LiPF_6_/[C_3_mPyr^+^][FSI^−^] electrolyte begins with cycling behaviour as reported previously^14^,^21^, with a periodic voltage response due to ambient temperature fluctuation. After 1,300 cycles, a period (*ca*. 250 h) of voltage instability is observed, culminating in cell death. Unpredictably, the cell is re-animated for 100 cycles (*ca*. 3,750–3,850 cycles) after a ‘dormancy' of 22 days (550 h) before permanently failing. This unusual behaviour suggests that failure is not due to a dendrite-induced short circuit, as this cannot be undone through continued galvanostatic cycling. The possibility that micro dendrites were formed during cycling, which created short circuits, but broke because of localized heating (that is, high resistances), thereby resurrecting the cell, was discounted because of no evidence of separator piercing. Figure 2b displays the *V–t* plot for a symmetrical cell containing LiAsF_6_/[C_3_mPyr^+^][FSI^−^], which cycled for 5,000 cycles reaching a polarization of 13 mV. This is attributed to a resistance decrease through repeated cycling forming a larger, and more porous surface^14^. Signs of voltage instability at various points, *ca*. 3,300, 3,950 and 4,700 cycles, are observed, representing morphology changes during SEI formation^36^. Symmetrical cells containing untreated lithium electrodes and LiFSI/[C_3_mPyr^+^][FSI^−^] electrolyte have also been reported to successfully achieve 5,000 cycles^2^. Therefore, these data illustrate that the electrochemical SEI built-up during cycling is undoubtedly electrolyte-dependent. For the case of LiAsF_6_ and LiFSI as previously reported, the pristine Li surface facilitates stable SEI formation and allows for effective cycling at a low current density of 0.1 mA cm^−2^ for up to 5,000 cycles; however, this is not the case when LiPF_6_ is used. As discussed later, specific pretreatment of the Li anode before cycling alleviates this phenomenon, and stable cycling can be achieved, which is completely independent of the Li salt used in the electrolyte.

To gain an insight as to why the use of one electrolyte and not another resulted in cell failure, a post-mortem analysis of the anode was undertaken for LiFSI, LiAsF_6_ and LiPF_6_ cells. In all cases, SEM images of the lithium electrode (Fig. 2c,e,g) exhibited no signs of dendritic growth of metallic Li from the surface; however, the quantity of surface species is higher than for the chemical pretreatment of the Li surface alone (Fig. 1), resulting in a rougher surface. The electrode surface of the cycled LiAsF_6_/[C_3_mPyr^+^][FSI^−^] cell (Fig. 2e) is completely fractured, and fresh Li^0^ nucleation now occurs at grain boundaries and steps similar to the LiFSI case (Fig. 2c). This surface-fracturing allows fresh lithium to be exposed, leading to further SEI formation. This is as expected for a mineral SEI, whereby pressure is released from within the SEI because of expansion/contraction via fracturing during lithium-plating/stripping processes. The gradual release of pressure prevents build-up and inherently inhibits dendritic growth^13^. The inset of Fig. 2e shows small nucleation sites that continue to form even near the latter stages of the 2,500-h charge–discharge cycle.

However, given the comparable nature of the SEI and lack of dendrite formation when LiPF_6_ is used, the brief re-animation indicates that cell failure is due to something other than an effect at the anode. Therefore, an investigation of the separator post cycling was undertaken. Interestingly, the SEM images (Fig. 2d,f,h) show electrolyte-breakdown products that have become detached from the lithium metal surface. For LiFSI (Fig. 2d) and LiAsF_6_ (Fig. 2f) these detached species are relatively sparse, and the porous nature of the separator is still quite visible, suggesting that the detachment of the SEI occurs at a slow rate. Figure 2d inset in fact shows that these deposits are located within the porous structure. This could suggest that bridging has occurred; however, these cells showed no significant drop in polarization for the experiment duration since the separator area blockage by the insulating SEI species is counteracted by the increase in lithium surface area through morphology changes. However, there is a clear discrepancy when the LiPF_6_ case is examined (Fig. 2h), as there is a significantly larger quantity of breakdown products observed within the pores that completely dominate the separator. In fact, the pores of the separator are no longer visible (see Supplementary Fig. 4 for SEM image of a pristine separator). Consequently, cell failure occurs because the rate of increase of the lithium surface area is lower than the rate of separator blockage, thus impeding Li^+^ flow and causing cell termination.

The FTIR measurements for electrochemically formed SEIs are included in Supplementary Table 4 and show that the chemical composition of the SEI after extensive cycling is relatively consistent with the measurements on the purely chemically formed SEI. However, the presence of cation-breakdown products is noticeably less for the LiFSI-based cell, with negligible responses in the ring-mode region (*ca*. 800–1,000 cm^−1^) and with fewer bands above 2,800 cm^−1^. The other chemistries, however, do have a larger abundance of cation products within the SEI layer. In fact, the cell composed of LiPF_6_ electrolyte exhibits a large response through the ring-mode region, indicating the extensive formation of cation-breakdown products, which may also contribute to the failure of this cell.

**Figure 3:**
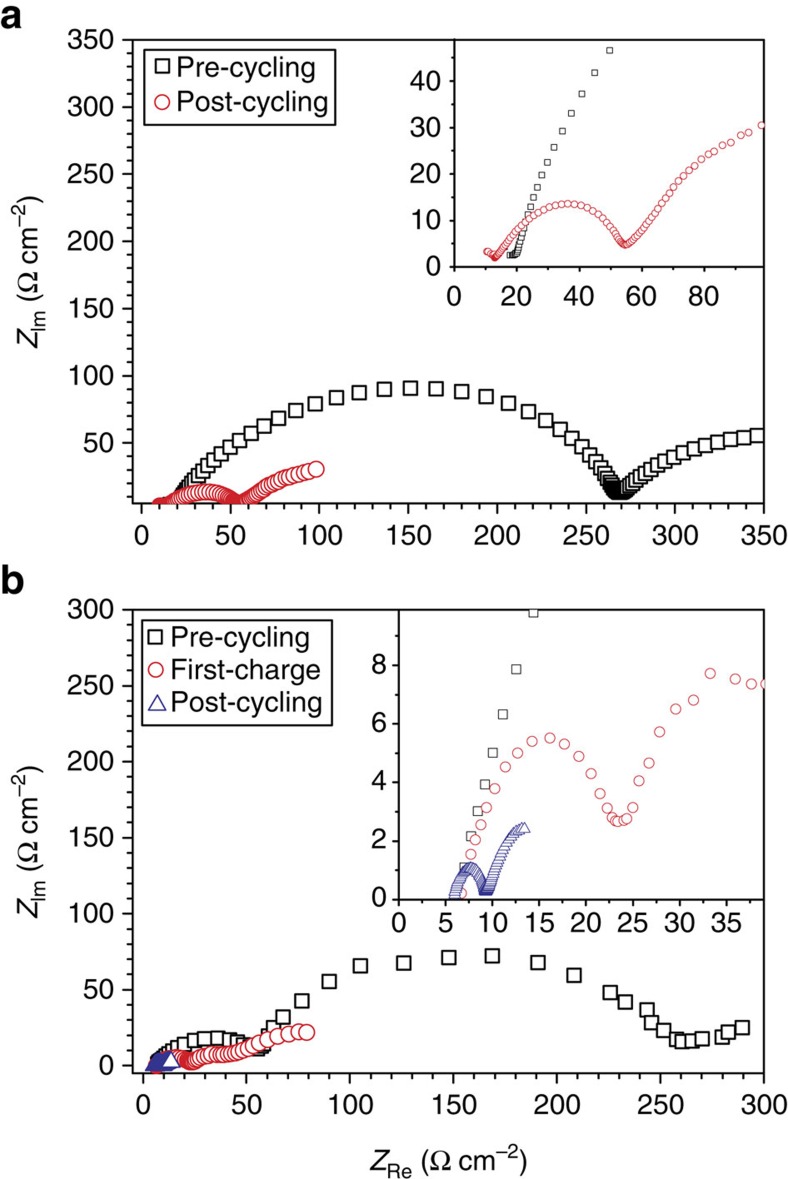
Electrochemical impedance spectroscopy of pristine and 12-day pretreated Li-symmetrical cells. Nyquist plots for a Li**-**symmetrical cell containing (**a**) LiAsF_6_/[C_3_mPyr^+^][FSI^−^] electrolyte before (black) and after 5,000 cycles (red) at 0.1 mA cm^−2^ and (**b**) 12-day pretreated Li electrode in LiFSI/[C_3_mPyr^+^][FSI^−^] electrolyte before (black) after one charge (red) and after 333 cycles (blue) at 1.0 mA cm^−2^.

Electrochemical impedance spectroscopy (EIS) was undertaken on Li-symmetrical cells before and after 5,000 cycles at a current density of 0.1 mA cm^−2^ to monitor any changes in the Li electrode impedance. EIS has been demonstrated to be highly informative for investigating battery electrodes^37^,^38^. For the pristine lithium cell using LiAsF_6_ salt, the interfacial resistance decreases from 251.5 to 51.3 Ω cm^−2^ after 5,000 cycles because of the morphological changes that occur in the SEI. Illustrated in Fig. 3a are typical Nyquist plots obtained for the LiAsF_6_ system before and after extensive cycling. This phenomenon was also observed for the LiFSI case where the impedance fell from 172.0 to 75.4 Ω cm^−2^ after 5,000 cycles (Supplementary Table 5).

### Analysis of pretreated electrodes at higher current density

**Figure 4:**
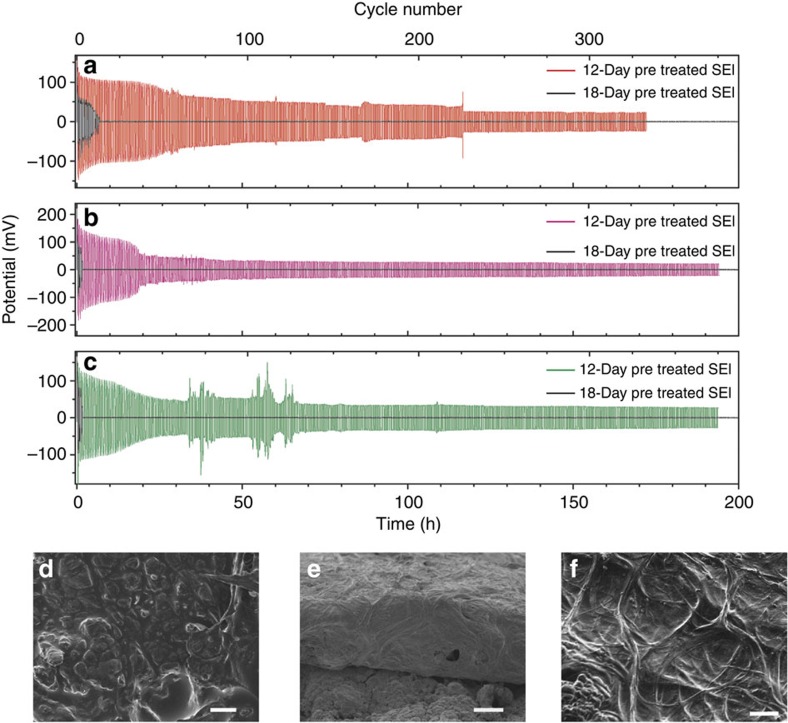
Comparison of pretreatment time for Li-symmetrical cells cycled at 1.0 mA cm^−2^. Voltage–time plots of Li-symmetrical cells using (**a**) Li|LiFSI/[C_3_mPyr^+^][FSI^−^]|Li, (**b**) LiPF_6_/[C_3_mPyr^+^][FSI^−^]|Li and (**c**) LiAsF_6_/[C_3_mPyr^+^][FSI^−^]|Li, after a period of 12 days (coloured) or 18 days (black). SEM micrographs of the Li|LiFSI/[C_3_mPyr^+^][FSI^−^]|Li cell components after 333 cycles highlighting the (**d**) lithium foil, (**e**) cross-section of the foil and (**f**) the separator material. Scale bars, 20 μm (**d**,**f**) and 100 μm (**e**).

The symmetrical Li|electrolyte|Li cell-cycling performance of the three pretreated electrodes and the associated electrolyte systems is presented in Fig. 4. The cells were cycled at 1.0 mA cm^−2^ following chemical pretreatment for 12- and 18-day time periods. The most obvious feature is that all the foils pretreated for 18 days begin cycling with large voltage instability, culminating in cell failure within just 6 h. Regardless of electrolyte composition, the SEI that is chemically formed over an 18-day period does not permit effective cycling of symmetrical cells at this current density. However, if the pretreatment is shortened to 12 days, the tailored SEI permits an exceptionally stable *V–t* profile for the entirety of the charge–discharge period. The cycling behaviour of the 12-day pretreated electrodes using LiFSI/[C_3_mPyr^+^][FSI^−^] (Fig. 4a) shows a gradual decrease in overpotential with repeated cycling where these overpotentials are generated via kinetic hindrances within the cell^21^. After a period of 225 cycles (*ca*. 128 h), the cycling behaviour of the symmetrical cell reaches a stable overpotential of 22.5 mV until the end of testing.

Cells of the 12-day pretreated LiPF_6_/[C_3_mPyr^+^][FSI^−^] system (failing if untreated, Fig. 2a) undergo a high degree of voltage instability within the first 40 h, reaching a steady and stable overpotential of 21 mV (Fig. 4b). Once this cell reaches a stable voltage, it exhibits the longest stability period for all three systems, displaying how an electrolyte of LiPF_6_ and [C_3_mPyr^+^][FSI^−^] can be practical for lithium metal batteries. The LiAsF_6_/[C_3_mPyr^+^][FSI^−^] system (Fig. 4c) also exhibits a stable overpotential in excess of 300 cycles. The initial *ca*. 50 h of cycling is followed by a *ca*. 30-h period of voltage instability and then by a stable cycling, reaching an overpotential of 28 mV until the end of testing. EIS data obtained for the symmetrical cells with the 12-day pretreated Li anodes for the LiFSI, LiPF_6_ and LiAsF_6_ cases also demonstrate a significant drop in the electrode impedance after 300 cycles at this higher current density of 1.0 mA cm^−2^ (Supplementary Table 5 and Supplementary Fig. 6). Typical Nyquist plots are shown for the LiFSI case in Fig. 3b. Promising cycling results for pretreated electrodes suggest that the application of ionic liquids may enable beneficial full-cell cycling. Furthermore, without pretreatment the electrodes do not cycle efficiently. Therefore, it appears that a smooth SEI formed via a chemical interaction of Li with the electrolyte, as indicated in Fig. 1d,h,l facilitates the formation of a stable and well-adhered SEI during electrochemical cycling. If this chemically formed SEI is rough, as shown after 18 days of interaction (Fig. 1e,i,m), it is not a good precursor to the formation of an SEI that is stable during cycling and it results in near-immediate cell failure.

The SEM micrographs of the lithium surface and separator after cycling are shown in Fig. 4d–f. They illustrate that stable SEIs are formed during electrochemical cycling of the pretreated anodes, in this case at a higher current density of 1 mA cm^−2^. Again, there is no evidence of dendritic growth on the Li surface or within the separator after extended cycling (Fig. 4d,f, respectively). Further evidence is provided in Fig. 4e, which shows a cross-section of the Li electrode that exhibits a smooth morphology devoid of any dendritic growth. SEM micrographs of other areas of the Li surface and separator are shown in Supplementary Fig. 5a,b to illustrate the homogeneity over the entire Li anode after cycling.

**Figure 5:**
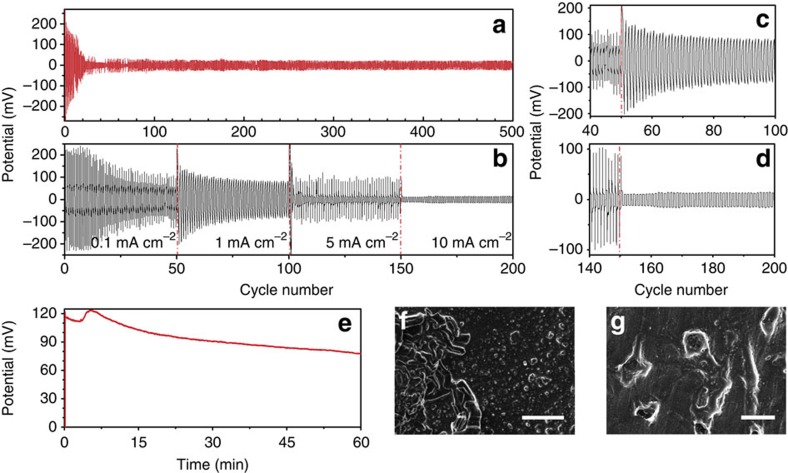
Characterization of Li|LiFSI/[C_3_mPyr^+^][FSI^−^]|Li cells after 12-day pretreatment. Cycled for 500 charge/discharge cycles at (**a**) 2.0 mA cm^−2^, (**b**) ramping 0.1 to 10 mA cm^−2^ for 50 cycles per current density and (**c**) highlighting the effect of ramping the current density from 0.1 to 1.0 mA cm^−2^ and (**d**) 5.0 to 10 mA cm^−2^. (**e**) The first charge/discharge of a symmetrical cell and the corresponding SEM images of the Li anode after the initial (**f**) plating (scale bar, 10 μm) and (**g**) stripping of lithium (scale bar, 50 μm).

Figure 5a shows the *V–t* plot of a symmetrical Li|LiFSI/[C_3_mPyr^+^][FSI^−^]|Li cell prepared with the aforementioned beneficial 12-day pretreatment time frame at a higher current density of 2 mA cm^−2^. Once again there is a period of larger polarization (>100 mV, 12 cycles) before the cell stabilizes (*ca*. 50 mV after 18 cycles) to 31 mV for the remainder of the cell's cycle lifetime of 150 h. A duplicate cell is cycled to observe the performance of the chemically formed SEI to a stepped current density, whereby the cell is cycled for 50 charge–discharge steps following a protocol of 0.1, 1.0, 5.0 and 10 mA cm^−2^ (Fig. 5b). The same behaviour of decreasing overpotential, after an initial sharp increase in the overpotential, is observed in the initial stages of cycling, and is compounded on the step-up of current density as highlighted within Fig. 5c. However, nearing a cycling time of *ca*. 80 h when stepping the current density to 10 mA cm^−2^ (Fig. 5d), the cell polarization is remarkably low and remains at an overpotential of *ca*. 11 mV. This indicates that there is rapid formation of the SEI on increasing the current density and a high enough supply of Li ions to the electrode that minimizes the possibility of any increases in electrode resistance as observed previously^11^. To gain more of an understanding of what occurs in the initial stages of electrochemical SEI formation on the pretreated lithium surface, a Li|LiFSI/[C_3_mPyr^+^][FSI^−^]|Li cell is charged at a current density of 1 mA cm^−2^ for 1 h and the *V–t* profile is shown in Fig. 5e. The SEM micrographs of the lithium electrode after plating and stripping of Li are displayed in Fig. 5f–g. Figure 5f shows the electrode after undergoing lithium plating, which consists of some large lithium deposits in areas where the chemical SEI pretreatment was not entirely effective. However, the areas where ideal chemical pretreatment occurred remain quite smooth. The opposing Li electrode after the stripping process, shown in Fig. 5g, exhibits pitting of the surface where the lithium metal has undergone electrodissolution. Supplementary Fig. 5c–f shows alternate areas of the cell components of this cell, highlighting the consistency of these observations. EIS data indicate that even after one charge the impedance of the Li electrode decreases substantially from 48.8 to 16.6 Ω cm^−2^ (Fig. 3b), which then reduces to 3.4 Ω cm^−2^ after more extensive cycling (Supplementary Table 5).

### Performance of full cells utilizing pretreated lithium anodes

**Figure 6:**
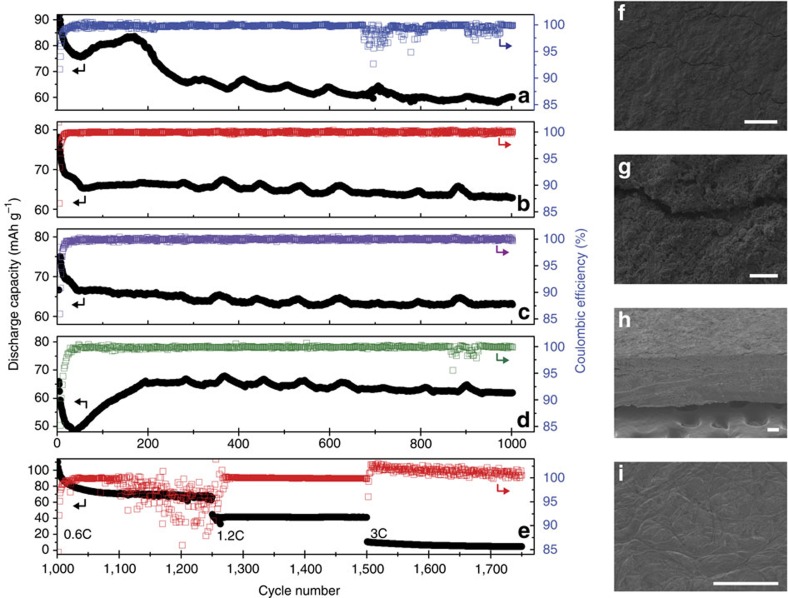
Electrochemical performance of LiFePO_4_ versus pristine and tailored lithium anodes after 12-day pretreatment with ionic liquid electrolytes. Discharge capacity and Coulombic efficiencies of Li|electrolyte|LiFePO_4_ full cells versus cycle number cycle at 1-c rate assembled with (**a**) pristine lithium and LiFSI|[C_3_mPyr^+^][FSI^−^] electrolyte. Discharge capacity and Coulombic efficiency for cells with LiFSI|[C_3_mPyr^+^][FSI^−^] electrolyte and pretreated with electrolytes composed of (**b**) LiFSI/[C_3_mPyr^+^][FSI^−^] (**c**) LiPF_6_/[C_3_mPyr^+^][FSI^−^] and (**d**) LiAsF_6_/[C_3_mPyr^+^][FSI^−^]. (**e**) Discharge capacity and Coulombic efficiency for the same LiFSI|[C_3_mPyr^+^][FSI^−^] cell in (**b**) after a shelf-life of 330 days. SEM micrographs of the LiFSI|[C_3_mPyr^+^][FSI^−^] cell (**f**), lithium anode (**g**), higher magnification (scale bar, 10 μm) of (**f**), (**h**) cross-section of (**f**) and (**i**) separator material. All other scale bars, 100 μm.

Secondary Li|electrolyte|LiFePO_4_ cells were also cycled (Fig. 6) to determine whether this chemically formed SEI could be beneficial for lithium metal batteries. On the basis of the symmetrical cell cycling, all electrodes were pretreated for 12 days. As a control, cells with pristine, untreated lithium metal were also cycled. The pristine lithium cell (Fig. 6a) displays a CE average of 99.6%; however, the CE plot is constantly undergoing fluctuation and becomes unstable after *ca*. 600 cycles where CE drops to 95.75%. This is not suitable for a commercial battery where technological demands require stability into thousands of cycles. Although the [C_3_mPyr^+^][FSI^−^] electrolyte has been shown to allow symmetrical cells to cycle effectively^11^,^12^ and for a long period (Fig. 4a) at high current density, this does not translate into stable cycling for full cells containing pristine lithium and LiFSI/[C_3_mPyr^+^][FSI^−^] electrolyte. The battery capacity after a period of stability decays to 80% of the initial capacity after *ca.* 600 cycles—commonly accepted as cell end of life.

The cells assembled with the pretreated SEI show an initial cell capacity decrease, which may be correlated to the 12-day pretreated symmetrical cell data shown in Fig. 4. The full cells begin cycling with an overpotential that relaxes as the cycling of the lithium lowers the cell resistance to a stable level in the order LiPF_6_, LiFSI and LiAsF_6_ after *ca*. 35, 60 and 130 cycles, respectively. This initial capacity fade for the LiFSI/[C_3_mPyr^+^][FSI^−^] (Fig. 6b) and LiPF_6_/[C_3_mPyr^+^][FSI^−^] (Fig. 6c) batteries is due to the anode up until the time periods highlighted above, and all further capacity changes are limited by the cathode (as is the case for the LiAsF_6_/C_3_mPyr^+^][FSI^−^] cell (Fig. 6d), reaching a plateau after *ca*. 200 cycles).

The average CEs for each of the pretreated cells are 99.96%, 99.93% and 99.42% for cells containing LiFSI, LiPF_6_ and LiAsF_6_, respectively. The minimum measured CE for the pretreated LiFSI/[C_3_mPyr^+^][FSI^−^] cell is an impressive 99.50%, reinforcing how positive an effect this facile chemical SEI formation method has on battery cycling. After an initial decrease in capacity during the first few cycles, the cell retains 95.4% capacity throughout the entirety of the 1,000 cycles.

The final CE values for the remaining LiPF_6_/[C_3_mPyr^+^][FSI^−^]- and LiAsF_6_/[C_3_mPyr^+^][FSI^−^]-pretreated cells, which also successfully cycled to 1,000 cycles are 99.02% and 99.89%, respectively. The capacity fade for these cells are *ca*. 5% with retention at 95.2% for the LiPF_6_ treatment, and 95.0% for the LiAsF_6_ treatment. A recent survey of the best systems reported in the literature (Table 2) demonstrates how this straightforward pretreatment method markedly improves battery-cycling performance. Ionic liquids have been used previously, and a table showing the performance of Li metal-based batteries using ionic liquids is presented as Supplementary Table 6. It is noteworthy that those studies are nowhere near comparable to the data presented here. Indeed, the full cell cycling data presented here are comparable if not better than many commercial Li ion batteries that were recently tested^25^.

After a quiescent period of 330 days, the Li|LiFSI/[C_3_mPyr^+^][FSI^−^]|LiFePO_4_ cell was again cycled, at stepped C-rates, to determine the effect of both the shelf-life and alternate current density (Fig. 6e). Initially, the cell demonstrated a large capacitance that stabilized after *ca*. 70 cycles (including three initial cycles at 0.1 C). Impressively, the full cell retains its charge that is equivalent to that previously measured on initial charge–discharge (Supplementary Table 7). The cell continues to provide close to its original capacity when initially cycled. Although an inoperative time of nearly 1 year required 70 cycles to achieve stability, this is quite comparable to the 40 cycles required for a fresh cell (Fig. 6b). From this point a capacity fade of only 9.6% is measured before stepping to the higher 1.2-C rate (7.3% fade). Figure 6e shows good cycling stability at a 1.2-C rate, with an associated CE greater than 98.5% for the entire 250 cycles, albeit this is reached after a brief period of morphology rearrangement as noted by the instability in the CE. The battery was then dismantled, and the Li anode demonstrated a compact SEI with some evidence of cracking (Fig. 6f,g). From the cross-sectional view (Fig. 6h), the SEI is compact and the thickness was, on average, 124 μm (Supplementary Fig. 7). As with the symmetrical cells, the separator did not exhibit any excessive build-up of material or dendrites, and the porous nature of the separator is still visible.

## Discussion

Stable cycling over a commercially acceptable time period of 2,000 h (1,000 cycles) was achieved at a 1-C rate, for lithium metal batteries without the formation of dendrites, thermal runaway or other detrimental characteristics commonly associated with lithium metal anodes. We herein have described a facile pretreatment methodology, which can be easily adopted by researchers and manufacturers with interest in lithium-metal batteries. As cycle life is related to the degree of electrolyte decomposition during cycling, this simple pretreatment of lithium anodes before cell fabrication ensures a reduction in electrolyte breakdown from SEI formation during the 1,000 cycles, thereby increasing cell performance and lifetime. This is highlighted for batteries with pristine, untreated lithium anodes, which do not achieve the same degree of success compared with pretreated cells. By pretreating lithium anodes the SEI must only undergo a rapid equilibration (for example, 35 cycles with LiPF_6_/[C_3_mPyr^+^][FSI^−^] pretreatment) before reaching unparalleled lithium metal plating/stripping in full-battery scenarios.

The implication of diverse SEI formation on lithium metal anodes using RTIL electrolytes highlights the importance of interaction time for robust SEI formation capable of being used in lithium-metal batteries for extensive and efficient cycling. The salt and RTIL compete during SEI formation, simultaneously contributing to a highly dynamic SEI comprising [C_3_mPyr]^+^, [FSI]^−^ and salt anion breakdown in the case of LiPF_6_, or negligible participation, for example, as from LiAsF_6_. Since adventitious water was minimized, the impact of salt anion impurities (that is, moist LiPF_6_-producing hydrofluoric acid (HF) was decreased. The major constituents of the mineral SEI formed after pretreatment are LiF, Li_2_CO_3_, LiSO_2_F, LiOH as well as cation-breakdown products via a Hofmann elimination mechanism.

Symmetrical cells cycled at 0.1 mA cm^−2^ with pristine lithium highlighted the significant dependence on the salt anion on cyclability and SEI formation during cycling. However, this effect can be completely eliminated in a facile manner via a 12-day pretreatment time, which provides an optimal SEI in all electrolytes permitting the ingress/egress of lithium ions and withstands the associated volume change when cycling at a higher current density of 1.0 mA cm^−2^. A longer pretreatment time of 18 days did not improve the performance, regardless of salt composition. The formation of a dense and rough SEI is not a beneficial seed layer for further electrochemical SEI formation, indicating the importance of contact time between the Li anode and the electrolyte before cycling of a full battery. These promising symmetrical cell measurements were confirmed in full cell cycling using a commercial cathode material. Each of the electrolyte systems studied herein has accomplished 1,000 charge–discharge cycles in a Li|electrolyte|LiFePO_4_ configuration, with no lower than 99% efficiency. The one drawback of this approach is that some cycling needs to be performed to achieve a stable response, which is likely to result in some electrolyte consumption, and efforts to reduce this effect are ongoing. However, full cells that were left dormant for nearly 1 year could be effectively cycled at different rates, with a performance comparable to freshly prepared cells. Therefore, this work sheds light on the pre-conditioning that is often utilized for commercial Li ion batteries before roll out. For a battery based on Li|electrolyte|LiFePO_4_ such pre-conditioning is greatly simplified, whereby Li only needs to be immersed in the relevant electrolyte for 12 days before cell fabrication.

## Methods

### Pretreatment of lithium anodes

Lithium metal foils (China Energy Lithium, 0.33 mm, 99%) were immersed in [C_3_mPyr^+^][FSI^−^] (Dai-Ichi Kogyo Seigaku, >99%) electrolyte mixtures containing 0.5 mol Kg^−1^ of a lithium salt for various time periods (4 h and 7, 12 and 18 days). These lithium salts include the following: LiFSI (Dai-Ichi Kogyo Seigaku, >95%), LiPF_6_ (Sigma, 98%) and LiAsF_6_ (Sigma-Aldrich, 98%). Lithium foils were cleansed of surface contaminants using a nylon brush under *n*-hexane (Merck). The ionic liquid was dried *in vacuo* using standard Schlenk line techniques at 100 °C for 12 h before storage and handling under an Ar atmosphere (less than 5 p.p.m. H_2_O and 1 p.p.m. O_2_). Karl Fischer titration analysis of the ionic liquids dried using this method indicated less than 10 p.p.m. H_2_O content. Lithium salts were dried according to literature reports^39^. On reaching the desired pretreatment time for submerged foils, they were removed and rinsed of any residual electrolyte using dimethyl carbonate (DMC) (Fluka) as reported previously^19^.

### Coin cell fabrication and cycling

Symmetrical Li|electrolyte|Li coin cells were fabricated using CR2032 metal jackets as reported previously^11^. The symmetrical cells underwent cyclic charge–discharge galvanostatic testing using a Maccor Series 4000 battery tester at ambient temperature (22.2±2 °C) with a low or high current density of 0.1 or 1.0 mA cm^−2^, respectively. Replicate coin cells were cycled for either hundreds or thousands of cycles as specified in the text. Charge and discharge times were 15 min each, with 30 s rest between cycles (a cycle is defined as a charging step followed by a discharge step). The electrolyte used in symmetrical cells containing pretreated electrodes is kept consistent with that used for the pretreatment. Lithium metal foils used throughout the experimental design were 10 mm in diameter and the amount of electrolyte measured was 60 μl.

Full Li|electrolyte|LiFePO_4_ cells were fabricated in the same manner as symmetrical cells, with the addition of the cathode material, and underwent galvanostatic testing using an electrochemical pretreatment and subsequent 1-C rate. A composite of LiFePO_4_ (90 wt%, Phostech), carbon black (2.5 wt%, Timcal Super P) and carboxymethyl cellulose binder (7.5 wt%, Sigma-Aldrich) was coated on aluminium foils using an automated pasting machine (MIT Corporation, Compact tape casting film coater, MSK-AFA-III) and, after drying, cut into disks of 10 mm diameter. Both LiFePO_4_ and separator materials (Solupor) were dried at 100 °C under vacuum for 12 h before being used in both symmetrical and full cells. All cell-material handling and cell fabrication were performed in an Ar-filled inert atmosphere glovebox operating at less than 5 p.p.m. H_2_O and 1 p.p.m. O_2_. Duplicate cells were cycled for all systems to ensure reproducibility of data. Before cycling, all cells underwent three formation cycles at a rate of C/10, followed by 1,000 cycles at a rate of 1 C (equivalent lithium current density of 1.25 mA cm^−2^) at 30 °C temperature. Impedance measurements were carried out using a Solartron 1255B Frequency Response Analyser, a signal amplitude <10 mV, between a frequency range of 100 kHz to 0.1 Hz.

### Surface characterization

Surface characterization was performed after rinsing and drying of material with DMC, as stated previously, after pretreatment or cycling was carried out. Dried electrodes were loaded and enclosed in a hermetically sealed attenuated total reflectance stage (Specac Golden Gate) to permit FTIR analysis. After enclosure, the stage was removed from the glove box and spectra were obtained using a spectrum 400 FTIR spectrometer (Perkin-Elmer). All spectra were recorded over 16 scans using 4-cm^−1^ resolution. SEM was carried out using a Philips XL-30 field emission gun scanning electron microscope. A specifically designed brass substrate facilitated imaging of samples transferred to the SEM via a hermetically sealed environmental chamber. Images were recorded with an accelerating voltage of 5 kV under vacuum of 1.7 × 10^−5 ^mbar. A Link ISIS (Oxford Instruments) energy-dispersive X-ray system was used to collect qualitative data of sample surfaces. A Microlab VG310F Spectrometer (Thermo) was used to collect XPS. Samples were introduced to the instrument using a dual layer glove bag (Sigma). Transfer of samples from the glove box to glove bag took place by storing a sample inside a Schott bottle within a heat-sealed aluminium laminate pouch (3 M Film). All XPS measurements were recorded after 7 days of electrolyte pretreatment to ensure that sufficient time had lapsed for SEI reduction products to be detected. It is at this 7-day period that minimal electrolyte could be observed via SEM; thus, any spectral data best describe structural SEI information rather than pore-trapped residues.

### Data availability

The data that support the findings of this study are available from the corresponding authors on request.

## Additional information

**How to cite this article:** Basile, A. *et al.* Stabilizing lithium metal using ionic liquids for long-lived batteries. *Nat. Commun.* 7:11794 doi: 10.1038/ncomms11794 (2016).

## Supplementary Material

Supplementary Information Supplementary Figures 1-7, Supplementary Tables 1-7, Supplementary Note 1 and Supplementary References

## Figures and Tables

**Table 1:** Summary of *ex situ* XPS for lithium metal pretreated with [C_3_mPyr^+^][FSI^−^] containing different lithium salts after a 7-day reaction period.

**SEI**	**Binding energy (eV)**						
**Component**	**Li** **1*s***	**C** **1*s***	**N** **1*s***	**O** **1*s***	**F** **1*s***	**S 2*p***	**P 2*p*/As 3*d***
*LiFSI/[C_3_mPyr^+^][FSI^−^] electrolyte pretreatment*							
C–N^+^		286.3	403.2				
NSO^−^			400.3	—		—	
C–C, C–H		285.0					
LiOH	55.2			532.4			
Li_2_CO_3_	55.2	289.8		533.4			
LiF	56.1				685.1		
Li_2_S	—					160.6	
LiSO_2_F				534.5	688.3	170.5171.9	
							
*LiPF_6_/[C_3_mPyr^+^][FSI^−^] electrolyte pretreatment*							
C–N^+^		286.4	403.2				
NSO^−^			400.5				
C–C, C–H		285.0					
LiF	—				685.3		
Li_2_CO_3_	55.0	288.4		533.0			
LiOH	55.0			531.9			
Li_2_S						160.5	
PF_*x*_					689.0		136.2
LiSO_2_F				534.3	688.2	170.2171.5	
							
*LiAsF_6_/[C_3_mPyr^+^][FSI^−^] electrolyte pretreatment*							
C–N^+^		286.4	403.2				
NSO^−^			400.2				
C–C, C–H		285.0					
Li_2_CO_3_	55.6	288.3		533.0			
LiF	56.7				685.6		
π–π*		291.3					
As							—
Li_2_S	—					160.2	
LiSO_2_F					688.7	170.0171.6	

XPS, X-ray photoelectron spectroscopy.

**Table 2:** Comparison and summary of recent literature reports on lithium metal battery cycling utilizing pretreatment methods.

**Cell treatment**	**Cell configuration**	**Cycle number**	**Cycling rate**	**Coulombic efficiency**	**Capacity fade**	**Reference**
Chemical interaction	Li|LiFePO_4_	1,000	1C	99.42–99.96%	5.0%	Present study
Carbon nanospheres on copper	[Li/CNS]|Li	150	0.25–1 mA cm^−2^	99.0%	—	Zheng *et al.*^22^
Graphene or BN layer via CVD and HT	Li|LiCoO_2_	50	0.5–5 mA cm^−2^	95.0%	—	Yan *et al.*^32^
PANI-CNT buffer layer	Li | [LiCoO_2_/CNT]	60	4C	—	14.0%	Zhang *et al.*^23^
Mechanical surface modification	Li|LiFePO_4_	150	C/2	—	15.0%	Ryou *et al.*^3^
Al_2_O_3_/polymer CPL	Li|LiCoO_2_	400	1C	99.8%	12.2%	Lee *et al.*^40^
LiFSI salt in carbonate solvent	Li|LiCoO_2_	50	0.5/0.2 C	—	7.0%	Han *et al.*^16^
Halogen reinforcement	Li|Li_4_Ti_5_O_12_	300	1C	—	—	Lu *et al.*^36^
Oxygen removal	LiTi_2_(PO_4_)_3_	1,000	C/8	—	10.0%	Luo *et al.*^41^
3D Copper foil	Li|Li-Cu	120	0.5 mA cm^−2^	98.5%	—	Yang *et al.*^42^
Hybrid POSS solid polymer electrolyte	Li|LiFePO_4_	50	C/2	>99%	n/a	Pan *et al.*^43^
LiNO_3_-ternary electrolyte	Li|Li-Cu	75	1 mA cm^−2^	94%	—	Zhao *et al.*^44^
Cu–graphene ‘drum' scaffold	Cu-C|Li	800	2 mA cm^−2^	93%	—	Zhang *et al.*^45^
Concentrated electrolyte	Li|LiFePO_4_	200	C/5	>92%	16.7%	Ma *et al.*^46^
Concentrated electrolyte	Li|FeF_2_	1,000	140 mA g^−1^	80 %	—	Gu *et al.*^47^
Concentrated LiNO_3_ in DMSO	Li|Li	90	0.2 mA cm^−2^	>80%	—	Togasaki *et al.*^48^
SiO_2_ hollow NCSE	Li|LiFePO_4_	200	C/5	—	<5.0%	Zhou *et al.*^49^
Concentrated electrolyte	Li-Cu	1,000	4 mA cm^−2^	98.4%	—	Qian *et al.*^50^

3D, three-dimensional; BN, boron nitride; CNT, carbon nanotube; CNS, carbon nanospheres; CPL, composite protective layer; CVD, chemical vapour deposition; DMSO, dimethylsulphoxide; HT, high temperature; NCSE, nanosphere composite solid–electrolyte; PANI, polyaniline; POSS, polyhedral oligomeric silsesquioxane.
