# Supplementary material for: Stabilizing lithium metal using ionic liquids for long-lived batteries
Source: Nat Commun. 2016 Jun 13;7:ncomms11794. doi: 10.1038/ncomms11794 (PMC4909938; doi:10.1038/ncomms11794)
Supplement: Supplementary Information — Supplementary Figures 1-7, Supplementary Tables 1-7, Supplementary Note 1 and Supplementary References [file ncomms11794-s1.pdf]

Supplementary Information

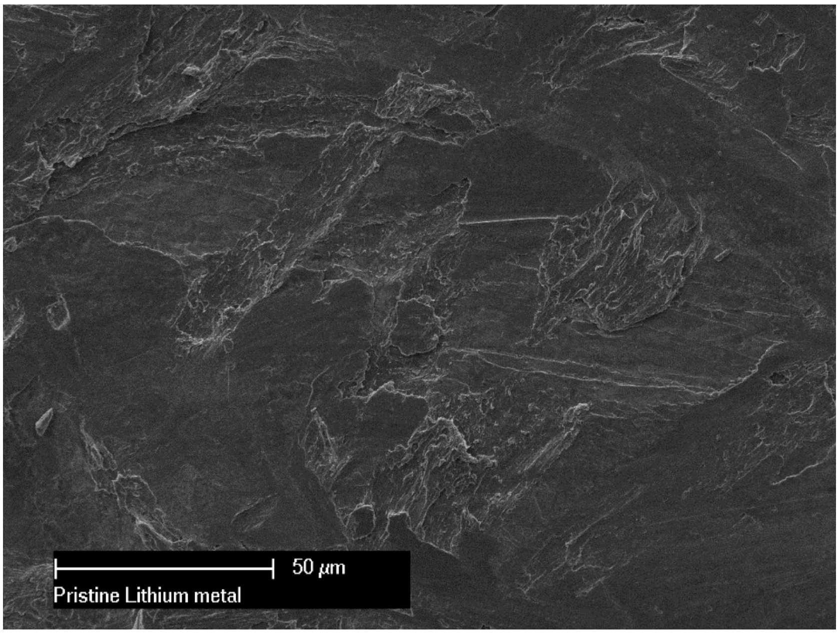

Supplementary Figure 1 | SEM image of a pristine lithium metal surface after polishing.

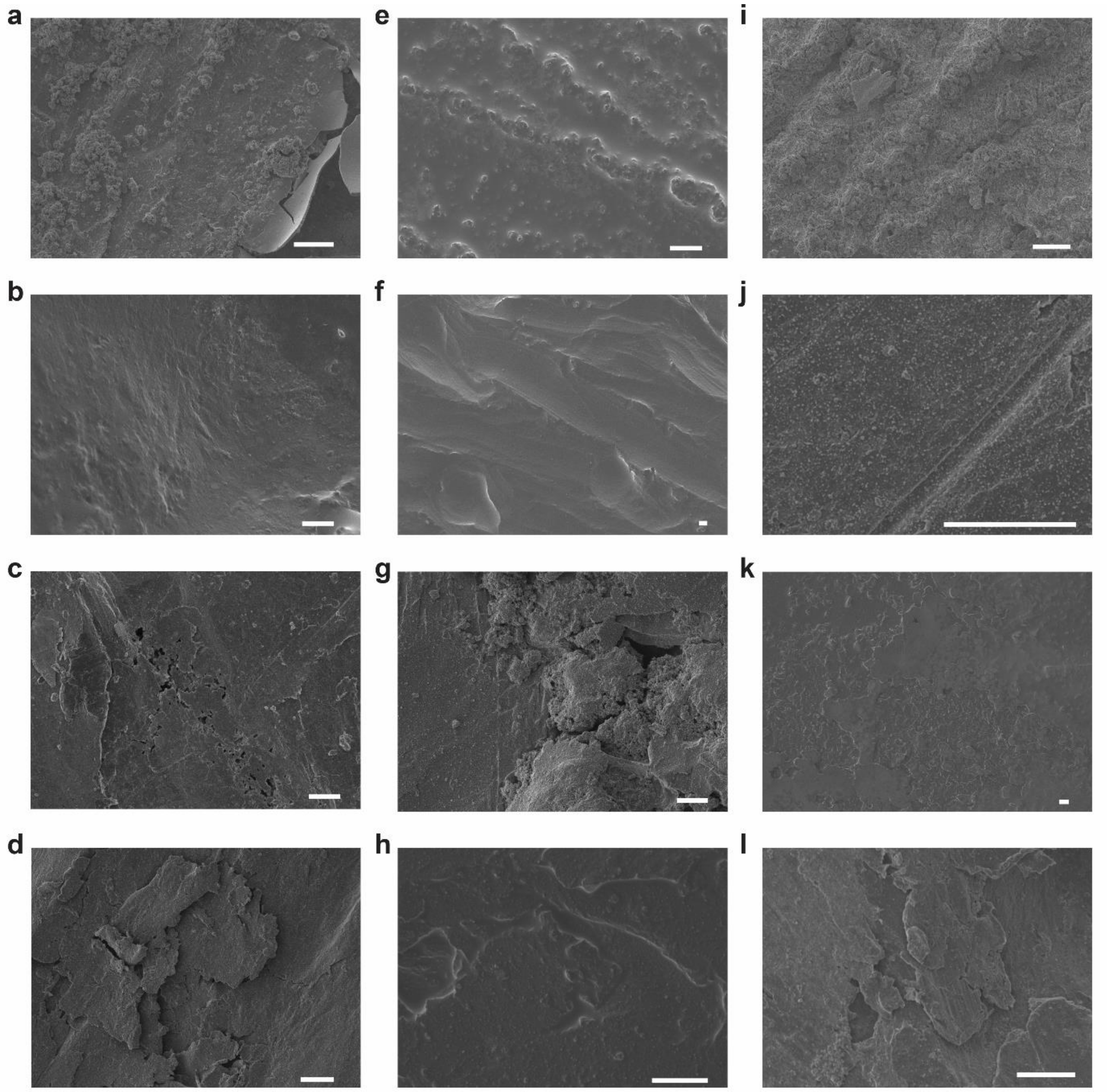

Supplementary Figure 2 | SEM micrographs of a lithium foil after chemical interaction with a LiFSI/[C_3mPyr$^+$][FSI^-] electrolyte after a, 4 days, b, 10 days, c, 14 days, d, 16 days. Using $LiPF_6$/[C_3mPyr$^+$][FSI^-] electrolyte after e, 4 days, f, 10 days, g, 14 days, h, 16 days. $LiAsF_6$/[C_3mPyr$^+$][FSI^-] electrolyte after i, 4 days, j, 10 days, k, 14 days, l, 16 days. Scale bars correspond to 10 µm.

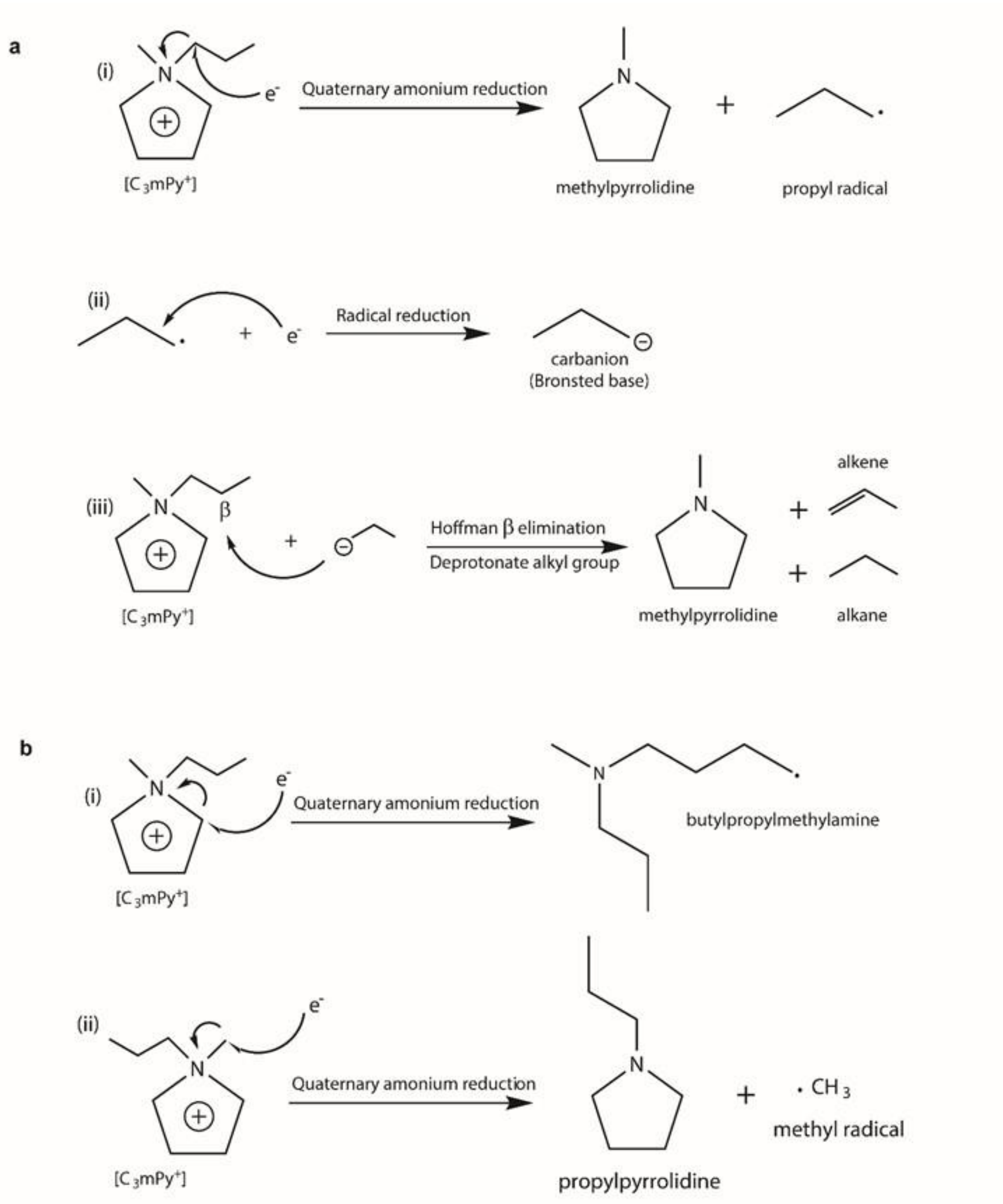

Supplementary Figure 3 | Schematic/mechanism for the SEI formed at the lithium metal surface via chemical interaction with ionic liquid electrolytes.

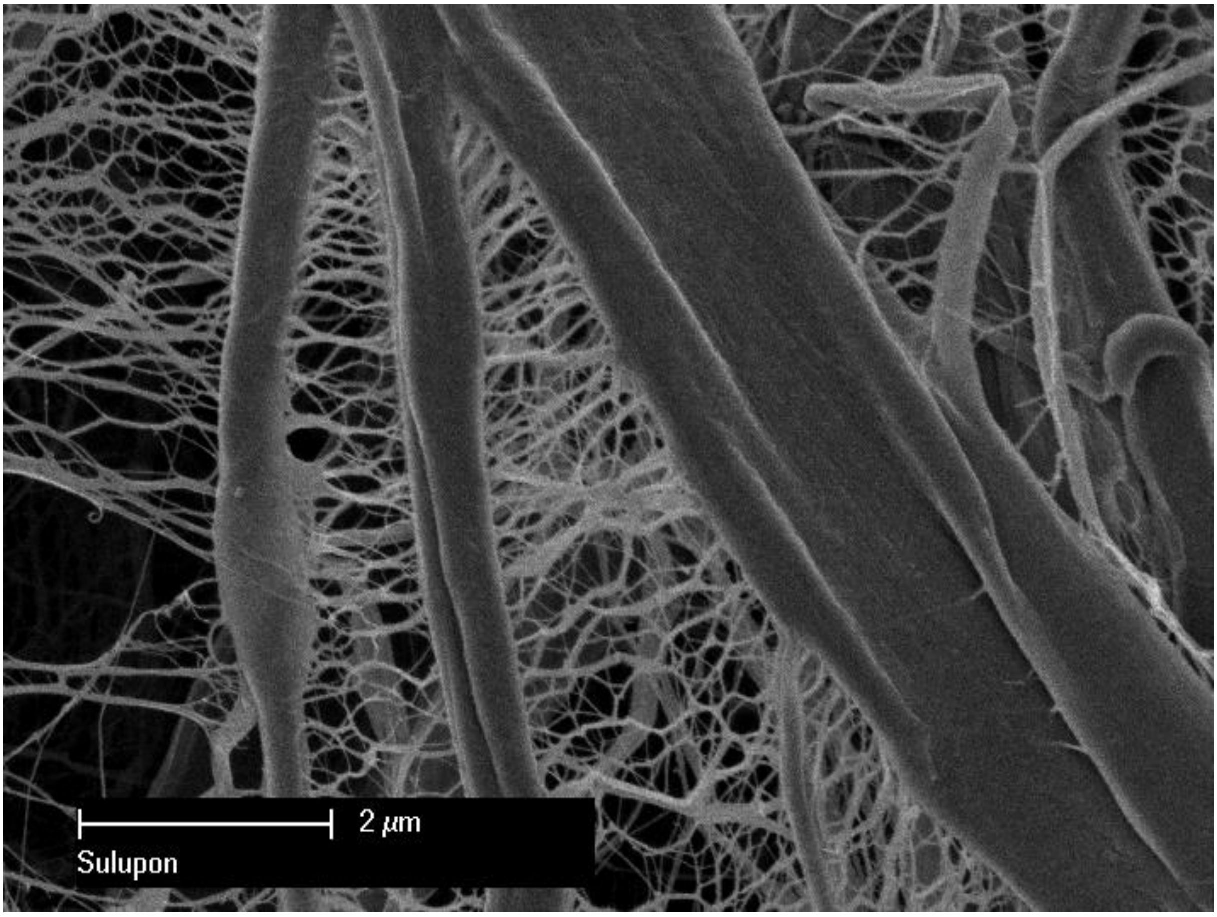

Supplementary Figure 4 | SEM image of a pristine separator. Reproduced with permission from Aust. J. Chem. 2013, 66, 252–261. (http://dx.doi.org/10.1071/CH12392)

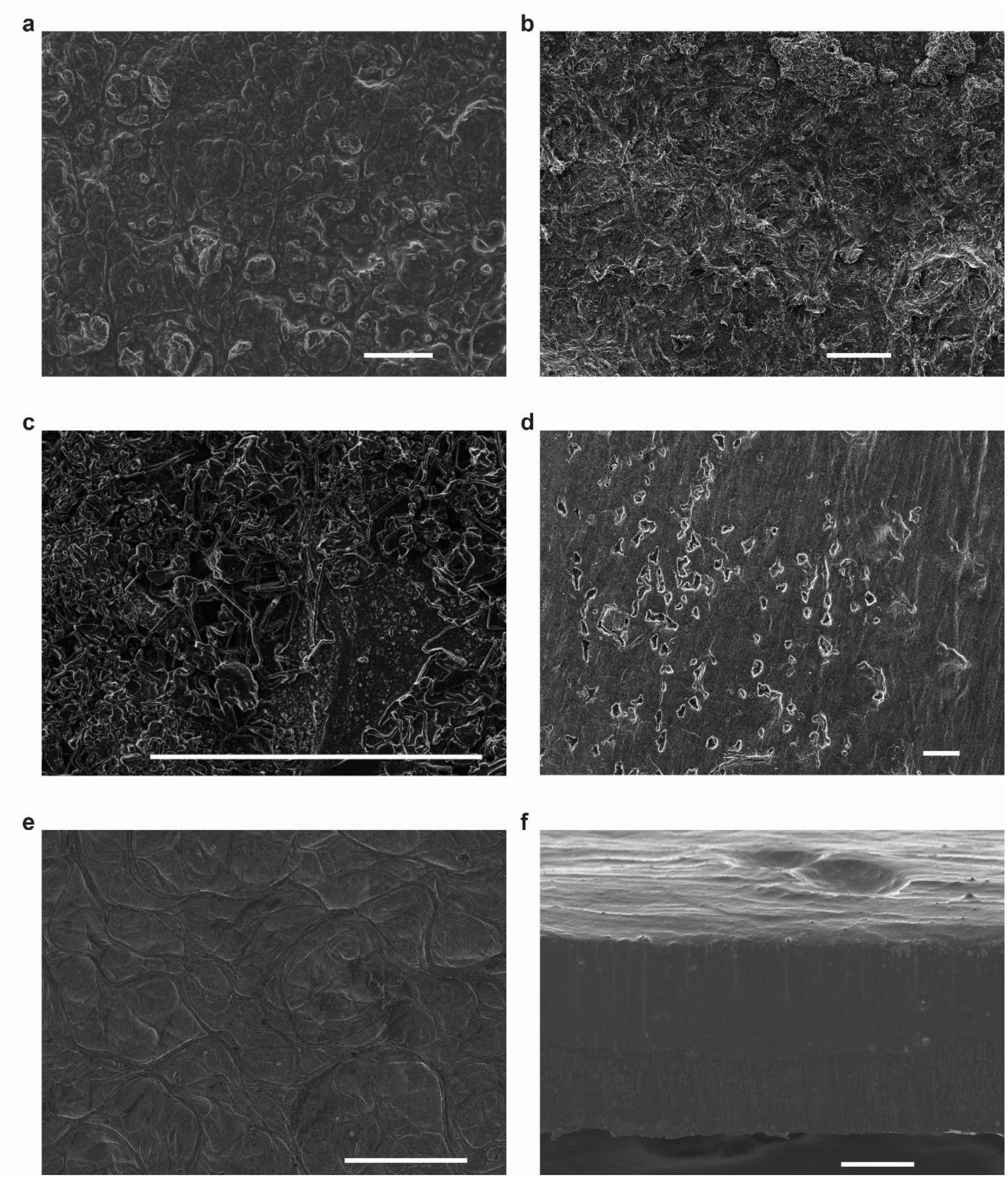

Supplementary Figure 5 | SEM micrographs of Li|LiFSI/[C_3mPyr$^+$][FSI^-]|Li symmetrical cells cycled with a current density of 1.0 mA cm^{-2} for various time periods. **a**, Alternate area of the lithium electrode after 333 cycles, **b**, separator material after 333 cycles, **c**, first electrodeposition of lithium, **d**, first electrodissolution of lithium, **e**, separator material after a single stripping/plating process, **f**, cross section of an electrode after the first stripping/plating process. Scale bars: 100 μm.

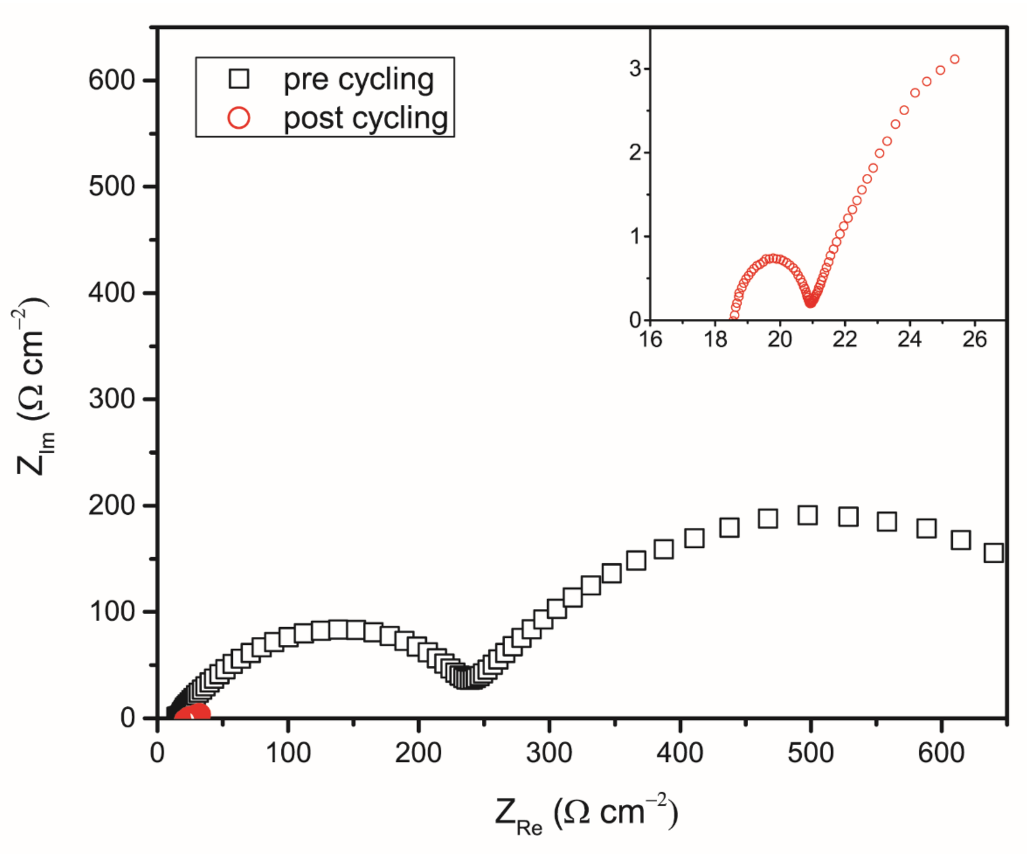

Supplementary Figure 6 | EIS measurement for the cell incorporating pretreated lithium foils over 12 days using $LiPF_6$/[C_3mPyr$^+$][FSI$^-$] prior to and post 300 cycles.

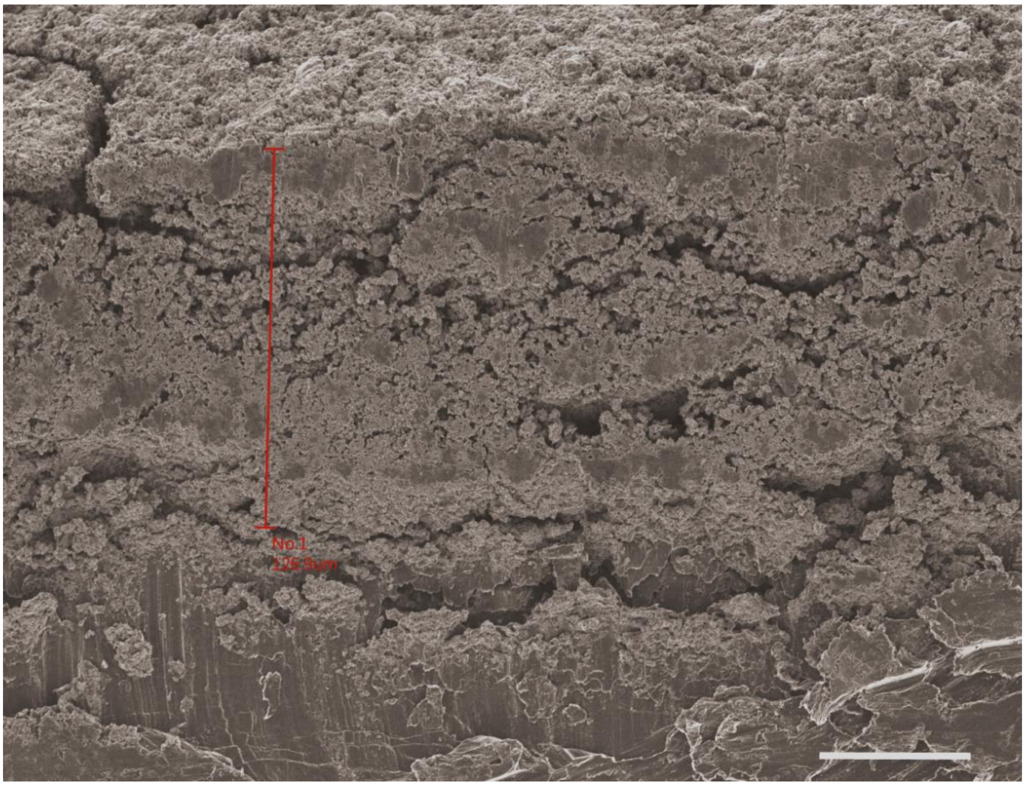

Supplementary Figure 7 | SEM micrograph of the cross section of the lithium anode after a total 1,750 cycles in a full cell configuration.

Supplementary Table 1 | Assignment of FTIR spectra for Li metal after interaction with LiFSI/[C_3mPyr^+][FSI^-] electrolyte.

**Assignment**	**Frequency (cm^{-1})**				**Reference(s)**
	4 hours	7 days	12 days	18 days	
νN-C, $\delta_s SO_2$	578 (s)	576 (s)	571 (s)	578 (s)	5, 6
$\delta_a SO_2$	611 (w)		619 (w)	611 (w)	5
νSF	656 (vw)			640	6
νSNS	755 (m)	751 (m)	745 (m)	753 (w)	5, 7
ρCH_2, Ring mode	835 (m)	828 (m)	830 (m)	834 (m)	8
Ring mode	886 (vw)		881 (sh)	891 (w.br)	5, 8
Ring mode		906 (vw)	901 (vw)		5, 8
Ring mode	940 (w)	938 (w)	939 (w)	940 (w)	5, 8
Ring mode	970 (vw)	972 (vw.br)	971 (vw)	971 (vw)	5, 8
Ring mode	1008 (w)	1003 (w)	1002 (w)	1015 (w)	5, 8
ν_aSNS		1058 (sh)	1061	1063	5, 6, 7
$\nu_a SO_2$-N-SO_2	1105 (s.br)	1105 (m)	1105 (s)	1101 (s)	5, 6, 7
νSO_2-N-SO_2	1190 (vs)	1175 (vs)	1175 (vs)	1189 (vs)	5, 6, 7
ρCH_2	1219 (m)	1216 (w)	1217 (m)	1218 (w)	8
$\nu_a SO_2$ (o.p.)	1366 (m)	1363 (s)	1360 (s)	1365 (m)	5, 6
νSO_2 (i.p.)	1387 (s)	1380 (s)	1379 (s)	1386 (s)	5, 6
δCH_2	1434 (vw)	1434 (w)	1434 (vw)	1433 (vw)	5, 8
νCH_2, νCH_3	1469 (m)	1462 (w)	1466 (m)	1472 (m)	5, 9
Li_2CO_3	1525 (vw.br)	1528 (vw.br)			1, 6, 10
amide, C=C		1641 (vw.br)			10
νCH_2, νCH_3 (3° amine)		2849 (w)			2, 11, 12
νC-CH			2893 (vw)	2889 (vw)	2
νC-CH		2921 (w)			2
νCH_2, νCH_2 (3° amine)			2947 (vw)	2944 (vw)	2, 5, 8
νCH_2	2978 (vw)	2978 (w.br)	2983 (vw)	2979 (vw)	5, 8
CH_2-N-CH_2 (2° amine)	3298 (w)	3298 (vw)		3298 (w)	13
νLi-OH				3677 (w)	2, 6, 10

w = weak, m = medium, s = strong, v = very, br = broad, sh = shoulder.
ν = stretching, δ = bending, τ = twisting, ω = wagging, ρ = rocking.
a = asymmetry, s = symmetry.

Supplementary Table 2 | Assignment of FTIR spectra for Li metal after interaction with $LiAsF_6$/[C_3mPyr$^+$][FSI$^-$] electrolyte.

Assignment	Frequency (cm^{-1})				Reference(s)
	4 hours	7 days	12 days	18 days	
νN-C, δ_sSO_2	578 (s)	583 (w)			5, 6
νLi-O~	595 (w.br)				6, 14, 15
δ_aSO_2	615 (vw)		627 (vw)	625 (vw)	5
νAs-F	703 (m)	702 (w)			16, 17, 18, 19
νSNS	754 (m)	751 (m)			5, 7
ρCH_2, Ring mode	834 (m)	830 (m)	827 (m)	830 (m)	8
Ring mode	880 (w)	870 (w.br)	867 (sh)	885 (vw.br)	5, 8
Ring mode	903 (vw)	907 (vw)			5, 8
Ring mode	939 (w)	939 (w)	937 (w)	940 (w)	5, 8
Ring mode	970 (w)	973 (vw)	967 (vw)		5, 8
Ring mode	1003 (w)	1002 (w)	997 (m)	1004 (m)	5, 8
ν_aSNS	1047 (w.br)	1042 (sh)	1048 (w)		5, 6, 7
ν_aSO_2-N-SO_2	1109 (s)	1102 (s)	1100 (s)	1100 (m)	5, 6, 7
νSO_2-N-SO_2	1188(vs)	1175 (vs)	1174 (vs)	1179 (vs)	5, 6, 7
ρCH_2	1218 (m)	1215 (m)	1215 (w)	1216 (m)	8
ν_aSO_2 (o.p.)	1365 (m)	1360 (m)	1359 (m)	1362 (m)	5, 6
νSO_2 (i.p.)	1387 (s)	1378 (m)	1379 (s)	1383 (s)	5, 6
δCH_2	1434 (vw)	1431 (w)	1429 (w)	1432 (vw)	5, 8
νCH_2, νCH_3	1472 (m)	1460 (w)	1460 (vw)	1464 (m.br)	5, 9
Li_2CO_3	1509 (w.br)	1509 (vw.br)	1502 (vw.br)	1494 (vw)	1, 6, 10
LiOH				1583(vw)	14, 20, 21
amide, C=C	1663 (m)	1629 (m)	1644 (m.br)	1652 (vw)	13
νCH_2, νCH_3 (3° amine)	2850 (w)	2850 (vw)	2850 (vw)	2852 (vw)	2, 11, 12
νC-CH	2889 (m)	2887 (vw)	2888 (vw)	2888 (w)	2
νC-CH	2918 (m)		2918 (vw)	2922 (vw)	2
νCH_2, νCH_2 (3° amine)	2947 (w)	2944 (vw)	2944 (vw)		2, 5, 8
νCH_2	2980 (vw)	2977 (vw)	2975 (vw)	2974 (vw)	5, 8
νLi-OH	3676 (m)	3674 (vw)		3675 (m)	6, 8, 10

Supplementary Table 3 | Assignment of FTIR spectra for Li metal after interaction with $LiPF_6$/[C_3mPyr^+][FSI^-] electrolyte.

Assignment	Frequency (cm^{-1})				Reference(s)
	4 hours	7 days	12 days	18 days	
νN-C, $\delta_s SO_2$		570 (s)	571 (s)	578 (s)	5, 6
νLi-O~			593 (w)		6, 14, 15
$\delta_a SO_2$	620 (vw)		619 (w)	611 (w)	5, 6
νSF	643 (vw)		643 (w)	641 (s)	6
νSNS	754 (m)	745 (m)	754 (m)	755 (w)	5, 7
ρCH_2, Ring mode	834 (m)	830 (m)	847 (m.sh)	840 (m)	8
$\nu LiPF_6$	850 (m.sh)		850 (m)	848 (m)	22
Ring mode	880 (w)	881 (m)	883 (sh)	892 (vw.br)	5, 8
Ring mode	916 (vw)	925 (m)	926 (vw)		5, 8
Ring mode	940 (w)			938 (w)	5, 8
Ring mode	968 (w)	970 (vw)	965 (w)	971 (vw)	5, 8
Ring mode	1005 (w)	1004 (w)	1002 (w)		5, 8
ν_aSNS	1041 (vw)	1040 (sh)	1044 (w)	1045 (sh)	5, 6, 7
$\nu_a SO_2$-N-SO_2	1110 (s)	1107 (s)	1114 (s)	1111 (s)	5, 6, 7
$\nu_s SO_2$, νLi-SO_2		1148 (s)			14
νSO_2-N-SO_2	1188 (vs)	1176 (vs)	1188 (vs)	1189 (vs)	5, 6, 7
ρCH_2	1219 (m)	1217 (m)	1218 (m)	1219 (m)	8
$\nu_a SO_{2\ (o.p.)}$	1365 (m)	1364 (m)	1364 (m)	1367 (m)	5, 6
$\nu SO_{2\ (i.p.)}$	1387 (s)	1381 (s)	1387 (s)	1387 (s)	5, 6
δCH_2	1434 (vw)	1432 (w)	1434 (vw)	1433 (vw)	5, 8
νCH_2, νCH_3	1469 (m)	1470 (m)	1469 (m)	1469 (m)	5, 8
Li_2CO_3	1505 (vw.br)	1513 (vw.br)	1510 (vw.br)		20, 1, 10
LiOH				1577	20, 14, 21
amide, C=C	1653 (w.br)	1628 (w)	1654 (w)		13
$\nu CH_{2\ (alkene)}$	1726 (w)				2, 13
$\nu CH_{2\ (alkene)}$	1754 (m)	1742 (vw)		1748 (w)	2, 13
νCH_2, $\nu CH_{3\ (3° amine)}$	2855 (vw)	2851 (vw)	2851 (w)	2854 (w)	2, 11, 12
νC-CH	2887 (w)	2857 (w)	2888 (w)		2
νC-CH		2920 (vw)	2923 (w)	2923 (w)	2
νCH_2, $\nu CH_{2\ (3° amine)}$			2947 (vw)	2946 (vw)	5, 2, 8
νCH_2	2978 (w)	2979 (m)	2977 (w)	2980 (w)	5, 8
CH_2-N-$CH_{2\ (2° amine)}$				3041 (vw)	13
CH_2-N-$CH_{2\ (2° amine)}$				3299 (vw)	13
νLi-OH	3677 (vw)			3677 (w)	8, 10, 20

Supplementary Table 4 | Assignment of FTIR spectra for pre-treated lithium metal after electrochemical rearrangement during symmetrical cell cycling with various salts within [C_3mPyr^+][FSI^-].

**Assignment**	**Frequency (cm^{-1})**			**Reference(s)**
	LiFSI	$LiPF_6$	$LiAsF_6$	
νN-C, δ_sSO_2	578 (s)	576 (s)	577 (s)	5, 6
δ_aSO_2	612 (s)	611 (sh)	610 (w)	5
δSNS	622 (s)	620 (m)	619 (w)	7
νSF	656 (vw)	653 (m)	654 (w)	6
Ring mode		726 (w.sh)		8
νSNS	755 (m)	753 (m)	755 (m)	5, 7
ρCH_2, Ring mode	835 (m)	833 (m)	833 (w)	8
Ring mode		880 (m)	881 (w)	5, 8
Ring mode		939 (w)	940 (w)	5, 8
Ring mode		974 (w)	975 (vw)	5, 8
Ring mode	1002 (w.sh)	1004 (m)	1004 (w)	5, 8
ν_aSNS	1064 (w.sh)	1060 (s)	1055 (w)	5, 6, 7
ν_aSO_2-N-SO_2	1105 (s.br)	1106 (m)	1108 (m)	5, 6, 7
νSO_2		1142 (m)		14
νSO_2-N-SO_2	1183 (vs)	1188 (vs)	1188 (s)	5, 6, 7
ρCH_2	1217 (m)	1216 (w.sh)	1218 (w)	8
νSO_2-N		1334 (m.sh)		14
$\nu_aSO_{2\ (o.p.)}$	1364 (m.sh)	1356 (s)	1365 (s)	5, 6
$\nu SO_{2\ (i.p.)}$	1383 (s)	1387 (s)	1387 (s)	5, 6
δCH_2		1433 (w.sh)	1439 (vw.sh)	5, 8
νCH_2, νCH_3	1460 (m)	1466 (m)	1470 (m)	5, 9
Li_2CO_3	1509 (vw.sh)	1503 (m.sh)	1500 (m)	1, 6, 10
amide, C=C		1624 (vw.br)	1620 (w)	10
$\nu CH_{2\ (3° amine)}$		2657 (vw)	2657 (w)	2
νCH_2, $\nu CH_{3\ (3° amine)}$	2881 (vw.sh)	2884 (w)	2883 (w)	2, 11, 12
Pyrrolidinium CH_2	2889 (vw)		2892 (vw)	2
νC-CH	2899 (vw)	2904 (vw)	2904 (w)	2
νC-CH	2917 (w)	2918 (vw)	2918 (vw)	2
νC-CH			2930 (w)	2
νCH_2, $\nu CH_{2\ (3° amine)}$	2961 (w.br)	2947 (w)	2947 (vw.sh)	2, 5, 8
νCH_2		2973 (w.br)	2973 (vw.br)	5, 8
CH_2-N-$CH_{2\ (2° amine)}$	3246 (vw.br)	3298 (vw.br)	3295 (vw.br)	13

Supplementary Table 5 | Li electrode impedance as a function of electrolyte, applied current density, cycling and electrode pretreatment.

Salt included in cell chemistry	Pre cycling (Ω cm^{-2})		Post cycling (Ω cm^{-2})	
	Solution	Interface	Solution	Interface
**0.1 mA cm^{-2} ×5000 cycles (Li|Li)<br>No ptretreatment**				
LiFSI	20.5	172.0	29.3	75.4
$LiPF_6$	12.0	169.3	-	-
$LiAsF_6$	17.8	251.5	13.0	51.3
**1.0 mA cm^{-2} ×300+ cycles (Li|Li)<br>12 day pretreatment**				
LiFSI	7.3	46.5	5.9	3.4
$LiPF_6$	17.2	221.6	23.5	3.1
$LiAsF_6$	25.9	150.9	15.5	4.0
**1.0 mA cm^{-2} ×1 charge step (Li|Li)<br>12 day pretreatment**				
LiFSI	6.4	48.8	6.8	16.6

Supplementary Table 6 | Performance of LMB using ionic liquids as electrolytes.

Cell configuration	Electrolyte	Cycle number	Cycling rate	Capacity fade	Reference
Li|$LiFePO_4$	0.7 *m* LiFSI $[C_3mPyr^+][FSI^-]$	38	1C	0%	23-26
Li|$LiFePO_4$	0.8 *m* LiTFSI $[C_4Pyr^+][TFSI^-]$	5	C/5	0%	24-28
Li|$LiCoO_2$	0.3 *m* LiTFSI $[EMIm^+][BF_4^-]$	50	C/8	14%	25-37
Li|ZrO_2-$LiCoO_2$	0.32 m LiTFSI $[DEME^+][TFSI^-]$	60	C/10	18%	26
Li|V_2O_5 nanoribbons	$[C_3mPyr^+][TFSI^-]$	50	2C	5%	27

Supplementary Table 7 | Comparison of cell capacity of newly fabricated and rested cell with the composition Li|LiFSI/[C_3mPyr$^+$][FSI$^-$]|$LiFePO_4$ according to cycle number. Cells cycled using a 1C rate unless otherwise indicated. *cycle at which stable capacity is reached.

	Cell capacity (mAh g^{-2})	
**Cycle Number**	**Newly fabricated**	**330 day shelf life**
1	78	110 (0.6)
40	67*	76
70	66	73*
100	66	71
200	66	66
300	65	42 (1.2C)
400	65	42 (1.2C)
550	64	8 (3C)
750	63	5 (3C)
1000	63	-

Supplementary Note 1 | FTIR study of solid electrolyte interface formation on lithium electrodes

Supplementary Table 1 shows the peak assignments for the lithium surface pre-treated using the LiFSI/[C_3mPyr$^+$][FSI$^-$] electrolyte. In particular the ring mode vibrations for the pyrrolidinium cation persist at greater intensity than those previously detected when lithium foil is pre-treated with neat [C_3mPyr$^+$][FSI$^-$].[1] This suggests that the increased concentration of [FSI]$^-$ may affect the mechanism of ring cleavage towards the propylpyrrolidine species, shown in **Supplementary Figure 3a**. The breakdown of pyrrolidinium cations has been suggested in recent publications.[2–4] The reduction taking place via a Hofmann β-elimination mechanism can result in three separate pathways.**Supplementary Figure 3b** highlights the two less favourable decomposition processes to form a radical at the quaternary ammonium, or methyl radical, via the same mechanism. The peak assignments in **Supplementary Table 1** do not include bands for alkene secondary products for methylpyrrolidine at 1728 and 1755 cm^{-1} further validating Hofmann β-elimination of N-methylpyrrolidinium toward propylpyrrolidine. This highlights the effect of the LiFSI in the electrolyte, affecting an alternate mechanism reported by Budi *et al.* when treating the lithium surface with neat [C_3mPyr$^+$][FSI$^-$].[1] With an increased [FSI]$^-$ concentration in this LiFSI/[C_3mPyr$^+$][FSI$^-$] electrolyte there is an associated rise in the intensity of bands at *ca.* 1106 and 1170 cm^{-1} suggesting a greater amount of anion breakdown products within the SEI. Peak assignments for those lithium foils pre-treated with either $LiAsF_6$/[C_3mPyr$^+$][FSI$^-$] or $LiPF_6$/[C_3mPyr$^+$][FSI$^-$] electrolyte are shown in **Supplementary Table 2** and **Supplementary Table 3** respectively.

Supplementary References

1. Budi, A. *et al.* Study of the Initial Stage of Solid Electrolyte Interphase Formation upon Chemical Reaction of Lithium Metal and N-Methyl-N-Propyl-Pyrrolidinium-Bis (Fluorosulfonyl) Imide. *J. Phys. Chem. C* **116,** 19789–19797 (2012).

2. Markevich, E. *et al.* In situ FTIR study of the decomposition of N-butyl-N-methylpyrrolidinium bis(trifluoromethanesulfonyl)amide ionic liquid during cathodic polarization of lithium and graphite electrodes. *Electrochim. Acta* **55,** 2687–2696 (2010).

3. Lane, G. H. Electrochemical reduction mechanisms and stabilities of some cation types used in ionic liquids and other organic salts. *Electrochimica Acta* **83,** 513–528 (2012).

4. Kroon, M. C., Buijs, W., Peters, C. J. & Witkamp, G.-J. Decomposition of ionic liquids in electrochemical processing. *Green Chem.* **8,** 241 (2006).

5. Howlett, P. C., Brack, N., Hollenkamp, A. F., Forsyth, M. & MacFarlane, D. R. Characterization of the lithium surface in N-methyl-N-alkylpyrrolidinium bis(trifluoromethanesulfonyl) amide room-temperature ionic liquid electrolytes. *J. Electrochem. Soc.* **153,** A595–A606 (2006).

6. Li, L. *et al.* Transport and Electrochemical Properties and Spectral Features of Non-Aqueous Electrolytes Containing LiFSI in Linear Carbonate Solvents. *Journal of The Electrochemical Society* **158,** A74 – A82 (2011).

7. Rey, I. *et al.* Spectroscopic and theoretical study of (CF3SO2) 2N-(TFSI-) and (CF3SO2) 2NH (HTFSI). *J. Phys. Chem. A* **102,** 3249–3258 (1998).

8. Bednarska-Bolek, B., Jakubas, R., Bator, G. & Baran, J. Vibrational study of the structural phase transition in bis (pyrrolidinium)-chloride-hexachloroantimonate (V) by infrared spectroscopy. *J. Mol. Struct.* **614,** 151–157 (2002).

9. Adebahr, J., Johansson, P., Jacobsson, P., MacFarlane, D. R. & Forsyth, M. Ab initio calculations, Raman and NMR investigation of the plastic crystal di-methyl pyrrolidinium iodide. *Electrochim. Acta* **48,** 2283–2289 (2003).

10. Morigaki, K. & Ohta, A. Analysis of the surface of lithium in organic electrolyte by atomic force microscopy, Fourier transform infrared spectroscopy and scanning auger electron microscopy. *J. Power Sources* **76,** 159–166 (1998).

11. Billes, F. & Geidel, E. Vibrational spectra and harmonic force fields of pyrrolidine derivatives: comparison between HF, MP2 and DFT force fields. *Spectrochim. Acta Part A Mol. Biomol. Spectrosc.* **53,** 2537–2551 (1997).

12. Degen, I. A. The Detection of the N-Methyl Group by Infrared Spectroscopy. *Appl. Spectrosc.* **23,** 239–241 (1969).

13. Dyer, J. R. *Applications of Absorption spectroscopy of Organic Compounds*. (Prentice-Hall, 1965).

14. Aurbach, D., Weissman, I., Zaban, A. & Chusid, O. Correlation between surface chemistry, morphology, cycling efficiency and interfacial properties of Li electrodes in solutions containing different Li salts. *Electrochim. Acta* **39,** 51–71 (1994).

15. Kominato, A. *et al.* Analysis of surface films on lithium in various organic electrolytes. *J. Power Sources* **68,** 471–475 (1997).

16. Aurbach, D. & Chusid, O. The use of in situ Fourier-transform infrared spectroscopy for the study of surface phenomena on electrodes in selected lithium battery electrolyte solutions. *J. Power Sources* **68,** 463–470 (1997).

17. Aurbach, D., Chusid, O., Weissman, I. & Dan, P. LiC(SO2CF3)3, a new salt for Li battery systems. A comparative study of Li and non-active metal electrodes in its ethereal solutions using in situ FTIR spectroscopy. *Electrochim. Acta* **41,** 747–760 (1996).

18. Ein-Eli, Y. *et al.* The dependence of the performance of Li-C intercalation anodes for Li-ion secondary batteries on the electrolyte solution composition. *Electrochim. Acta* **39,** 2559–2569 (1994).

19. Aurbach, D., Weissman, I., Schechter, A. & Cohen, H. X-ray Photoelectron Spectroscopy Studies of Lithium Surfaces Prepared in Several Important Electrolyte Solutions. A Comparison with Previous Studies by Fourier Transform Infrared Spectroscopy. *Langmuir* **12,** 3991–4007 (1996).

20. Aurbach, D., Gofer, Y. & Langzam, J. The correlation between surface chemistry, surface morphology, and cycling efficiency of lithium electrodes in a few polar aprotic systems. *J. Electrochem. Soc.* **136,** 3198–3205 (1989).

21. Parker, S. F., Refson, K., Bewley, R. I. & Dent, G. Assignment of the vibrational spectra of lithium hydroxide monohydrate, LiOH.H2O. *J. Chem. Phys.* **134,** 084503 (2011).

22. Geniès, S., Yazami, R., Garden, J. & Frison, J. C. SEM and FT-IR characterization of the passivation film on lithiated mesocarbon fibers. *Synth. Met.* **93,** 77–82 (1998).

23. Guerfi, a., Duchesne, S., Kobayashi, Y., Vijh, a. & Zaghib, K. LiFePO4 and graphite electrodes with ionic liquids based on bis(fluorosulfonyl)imide (FSI)- for Li-ion batteries. *J. Power Sources* **175,** 866–873 (2008).

24. Fernicola, A., Croce, F., Scrosati, B., Watanabe, T. & Ohno, H. LiTFSI-BEPyTFSI as an improved ionic liquid electrolyte for rechargeable lithium batteries. *J. Power Sources* **174,** 342–348 (2007).

25. Seki, S. *et al.* Lithium Secondary Batteries Using Modified-Imidazolium Room-Temperature Ionic Liquid. *J. Phys. Chem. B* **110,** 10228–10230 (2006).

26. Seki, S. *et al.* Highly reversible lithium metal secondary battery using a room temperature ionic liquid/lithium salt mixture and a surface-coated cathode active material. *Chem. Commun. (Camb).* **2,** 544–545 (2006).

27. Chou, S. L. *et al.* High capacity, safety, and enhanced cyclability of lithium metal battery using a v2O5 nanomaterial cathode and room temperature ionic liquid electrolyte. *Chem. Mater.* **20,** 7044–7051 (2008).
